# Gastrointestinal stem cells in health and disease: from flies to humans

**DOI:** 10.1242/dmm.024232

**Published:** 2016-05-01

**Authors:** Hongjie Li, Heinrich Jasper

**Affiliations:** 1 Buck Institute for Research on Aging, 8001 Redwood Boulevard, Novato, CA 94945-1400, USA; 2 Department of Biology, University of Rochester, River Campus Box 270211, Rochester, NY 14627, USA

**Keywords:** Intestine, Regeneration, Stem cells, *Drosophila*, Cancer, Metaplasia, Dysplasia

## Abstract

The gastrointestinal tract of complex metazoans is highly compartmentalized. It is lined by a series of specialized epithelia that are regenerated by specific populations of stem cells. To maintain tissue homeostasis, the proliferative activity of stem and/or progenitor cells has to be carefully controlled and coordinated with regionally distinct programs of differentiation. Metaplasias and dysplasias, precancerous lesions that commonly occur in the human gastrointestinal tract, are often associated with the aberrant proliferation and differentiation of stem and/or progenitor cells. The increasingly sophisticated characterization of stem cells in the gastrointestinal tract of mammals and of the fruit fly *Drosophila* has provided important new insights into these processes and into the mechanisms that drive epithelial dysfunction. In this Review, we discuss recent advances in our understanding of the establishment, maintenance and regulation of diverse intestinal stem cell lineages in the gastrointestinal tract of *Drosophila* and mice. We also discuss the field's current understanding of the pathogenesis of epithelial dysfunctions.

## Abstract (precis)

***Drosophila* Collection:** This Review highlights recent findings on intestinal stem cell (ISC) diversity in the GI tract of *Drosophila*, focusing on the role of ISCs in healthy and diseased conditions, and drawing parallels to vertebrate GI stem cells.

## Introduction

The gastrointestinal (GI) tract of most metazoans, including that of humans, is lined by a series of highly compartmentalized epithelia that perform localized functions. These epithelia also share certain characteristics that support interactions with commensal bacteria (see Box 1 for Glossary), enhance immune responses to infections and maintain the barrier function of the intestine. These epithelia undergo regeneration in homeostatic conditions as well as in response to tissue damage. During recurring regenerative episodes, and for the lifetime of the animal, the functional diversity of newly formed intestinal cells has to be sustained – an achievement that is only beginning to be understood, via the use of animal models (Barker et al., 2010a; Buchon et al., 2013b; Li et al., 2013a; Marianes and Spradling, 2013; Strand and Micchelli, 2011).

Conditions that negatively impact epithelial compartmentalization can have substantial deleterious consequences. In the human GI tract, for example, the development of epithelial metaplastic lesions (see Box 1) can place individuals at a high risk of developing intestinal cancer (Slack, 2007). In these lesions, one differentiated cell type is replaced by a cell with a different identity. One example is Barrett's metaplasia (see Box 1), in which the esophageal squamous epithelium acquires properties that are reminiscent of the gastric or intestinal columnar epithelium. This transformation has been associated with acid reflux disease and is believed to be a cause of esophageal adenocarcinomas (Falk, 2002; Hvid-Jensen et al., 2011). Dysplasia (see Box 1), another type of epithelial lesion that commonly affects the human GI tract, is characterized by aberrant cell proliferation and differentiation. Dysplastic changes are often found at later stages during epithelial carcinogenesis than are metaplasias, and eventually lead to invasive carcinoma (see Box 1) (Correa and Houghton, 2007; Ullman et al., 2009). Much remains to be learnt about intestinal metaplasias and dysplasias, not least because of their clinical significance, such as the exact cellular origins of metaplastic cells and the molecular pathways that underpin epithelial dysfunction. Because intestinal epithelia are regenerated by local intestinal stem cell (ISC) populations, which have been characterized in the GI tract of flies and mice in the past decade (Barker et al., 2007; Micchelli and Perrimon, 2006; Ohlstein and Spradling, 2006), deregulation of these stem cell functions, including proliferation and differentiation, has been associated with metaplastic and dysplastic lesions. To better understand the molecular changes underlying epithelial carcinogenesis, it is thus crucial to pinpoint the mechanisms that regulate ISC function.

In this Review, we highlight recent insights into the maintenance of GI compartmentalization and regulation of diverse stem cell lineages, focusing on advances made by analysis of the GI tract of *Drosophila* and mice. We then summarize findings about signaling pathways that control these stem cell functions, drawing parallels between the fly and mammalian systems. Finally, we discuss how these findings inform our current understanding of the pathogenesis of epithelial dysfunctions that can predispose humans to cancer.

## GI tract compartmentalization and stem cell lineages

The GI tract of most metazoans is highly compartmentalized in terms of morphology and function, and regional epithelial subtypes are continuously regenerated by local stem cell populations. Both in the *Drosophila* and mouse GI tracts, studies are underway to characterize the identity, function and regulation of regionally specified stem cell populations and stem cell lineages. Below, we provide an overview of *Drosophila* and mammalian GI tract morphologies and their respective stem cell populations.

### The *Drosophila* GI tract

**Fig. 1.**
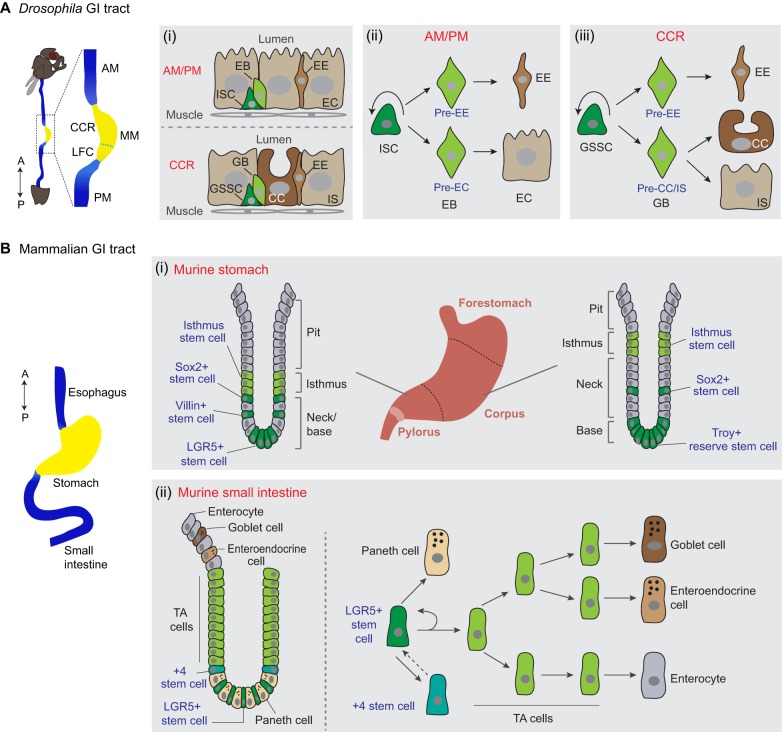
***Drosophila* and mammalian gastrointestinal (GI) tracts and associated stem cell lineages.** (A) A schematic of the *Drosophila* GI tract [‘A’, anterior, top; ‘P’, posterior, bottom), focusing on the midgut, which is divided into three main regions: the anterior midgut (AM), the middle midgut (MM) and the posterior midgut (PM). The MM contains an acidic stomach-like copper cell region (CCR) and a large flat cell region (LFC). (i) Organization of epithelial cells in the AM and PM (top), and the CCR (bottom). (ii) In the AM or PM, intestinal stem cells (ISCs) divide asymmetrically, giving rise to a new ISC and either a pre-enterocyte (pre-EC) enteroblast (EB), which will differentiate into an enterocyte (EC), or a pre-enteroendocrine-cell (pre-EE) EB, which will differentiate into an enteroendocrine cell (EE). (iii) In the CCR, gastric stem cells (GSSCs) undergo similar asymmetric division, giving rise to a new GSSC and two types of gastroblast (GB), which will differentiate into either a copper cell (CC), an interstitial cell (IS) or an EE. (B) The mammalian GI tract consists of an esophagus, stomach, small intestine and large intestine (not shown here) (anterior, top; posterior, bottom). (i) The murine stomach divides into three regions: forestomach, corpus and pylorus. The architecture of the gland unit, including the crypt, in the stomach corpus (right) and pylorus (left) is shown. Different stem cell populations (blue text) have been characterized, but exact stem cell lineages and cell hierarchies are unclear in these regions. (ii) In the murine small intestine, there are two main stem cell populations: +4 stem cells and LGR5+ stem cells, which can replace each other under certain conditions. A schematic of the crypt is shown on the left, and cell lineages are shown on the right. TA cells, transit amplifying cells.

The *Drosophila* GI tract is lined by a series of pseudostratified monolayer epithelia, which are surrounded by visceral muscle cells. Morphologically, the midgut, which is the main and best characterized part of the fly GI tract, is subdivided into the anterior midgut (AM), the middle midgut (MM) and the posterior midgut (PM) by two main constrictions (Fig. 1A). The MM contains a stomach-like copper cell region, which produces gastric acid, and a large flat cell region, the function of which is not well understood. Two recent studies have further divided the GI tract into 10-14 regions based on more detailed characterizations of morphological and molecular landmarks (Buchon et al., 2013b; Marianes and Spradling, 2013). ISCs are found in each of these compartments and can regenerate to give rise to all intestinal epithelial cell types (Table 1) (Buchon and Osman, 2015).

**Box 1. Glossary**

**Carcinoma:** a type of cancer that develops from epithelial cells.

**Commensal bacteria:** normal microorganisms living on the surface or within the body of their host in a symbiotic relationship in which the host is benefited or unaffected.

**Dysplasia:** an abnormality of development or an epithelial anomaly of growth and differentiation.

**Gastric atrophy:** a condition in which the stomach shrinks owing to loss of gastric glandular cells.

**Gastritis:** inflammation of the lining of the stomach.

***Lgr5*-driven inducible Cre:** a technique used in mice in which Cre recombinase expression can be induced by a drug, most commonly tamoxifen, in LGR5-expressing cells and catalyzes DNA recombination at *loxP* sites either to delete one gene or to induce reporter gene expression for lineage tracing.

**Metaplasia:** replacement of one differentiated cell type by another mature differentiated cell type.

**Niche:** stem cell niche is a microenvironment that interacts with stem cells to regulate their function.

**Organoids:** three-dimensional multicellular organs cultured *in vitro*.

**Squamocolumnar junction:** the region connecting the esophageal squamous epithelium and the gastric columnar epithelium in the gastrointestinal tract.

**Squamous keratinocytes:** cells found in the mucosa of the mouth and esophagus, as well as the corneal, conjunctival and genital epithelia, forming tight junctions and playing a role in immune system function.

**Teratomas:** a tumor with tissue or organ components resembling normal derivatives of more than one germ layer.

**Trans-differentiation:** direct conversion of one mature somatic cell to another mature somatic cell without going through an intermediate pluripotent state.

**Table 1.**
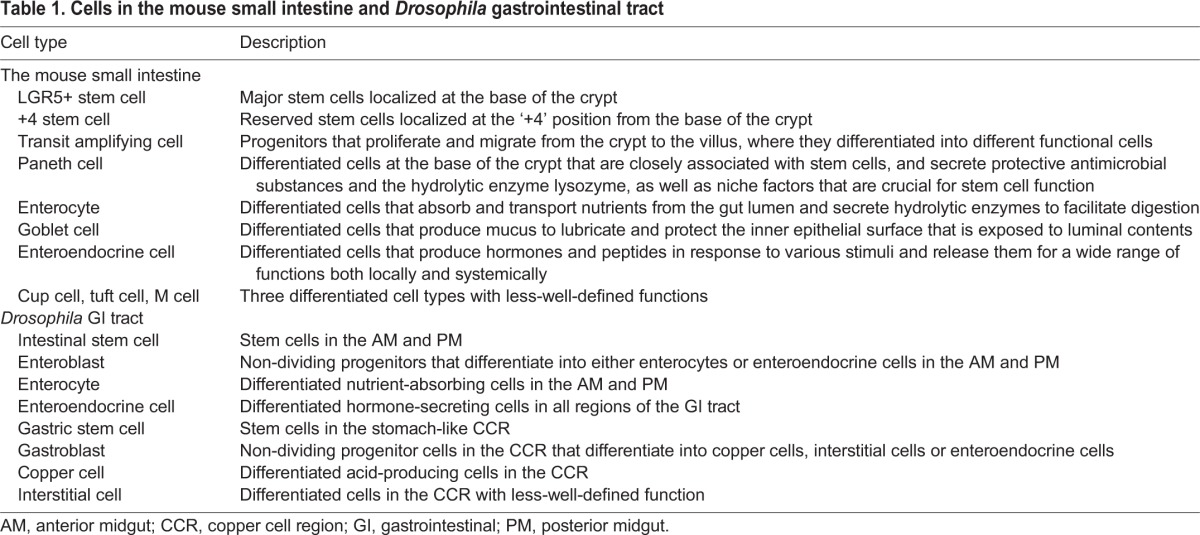
**Cells in the mouse small intestine and *Drosophila* gastrointestinal tract**

The ISC lineage was first characterized in the PM by two groups simultaneously (Micchelli and Perrimon, 2006; Ohlstein and Spradling, 2006) (Fig. 1A). Numerous studies subsequently characterized the regulation of ISCs and their lineage relationship to their progeny. As discussed later, a multitude of local, paracrine and systemic signals and signaling pathways that control ISC proliferation and differentiation have been identified, and changes in ISC function during tissue damage and aging have been described (Biteau et al., 2011; Buchon et al., 2013a; Buchon and Osman, 2015; Jiang and Edgar, 2011; Lemaitre and Miguel-Aliaga, 2013). ISCs [characterized by expression of Escargot (Esg+) and Delta (Dl+)] constitute the majority of cells capable of mitosis in the PM. During regenerative episodes, ISCs in the PM undergo asymmetric division to give rise to precursor cells, called enteroblasts [which have the marker profile Esg+, Su(H)GBE+, Dl−]. Enteroblasts further differentiate into either absorptive enterocytes [ECs; Pdm1+ (also called nubbin or POU domain protein 1)] or secretory enteroendocrine cells [EEs; Prospero (Pros+)] (Micchelli and Perrimon, 2006; Ohlstein and Spradling, 2006, 2007). Recent studies have refined our understanding of EE specification (Fig. 1A) (Beehler-Evans and Micchelli, 2015; Biteau and Jasper, 2014; Guo and Ohlstein, 2015; Wang et al., 2015a; Zeng and Hou, 2015). *In vivo* lineage-tracing methods suggest that these cells are generated from pre-committed Pros-expressing ISCs, and not, as previously described, as an alternative to EC differentiation from a common enteroblast cell. EE regeneration from pre-committed Pros+ ISCs is limited by Slit, an EE-derived ligand for the roundabout 2 (Robo2) receptor. Slit binds Robo2 on ISCs, setting up a negative-feedback loop from differentiated EEs that limits further production of these cells (Biteau and Jasper, 2014). A recent study shows that Ttk69, a BTB (broad complex, tramtrack and bric a brac)-domain-containing transcriptional repressor, also plays an important role in EE specification, presumably in parallel to Slit-Robo signaling (Wang et al., 2015a). Another study has further analyzed EE cell diversity and found that Su(H)GBE+ (Notch active) enteroblasts can give rise to class II EE cells, in addition to ECs (Beehler-Evans and Micchelli, 2015).

In the *Drosophila* copper cell region, which shares some similarity to the stomach in vertebrates, gastric stem cells (GSSCs; Esg+ and Dl+) generate three different cell types: copper cells, which secrete hydrochloric acid and are characterized by the marker profile Defective proventriculus (Dve)+/Labial(high)/Cut+; interstitial cells, defined by the profile Dve+/Labial(low)/Cut−; and Pros-expressing EEs (Strand and Micchelli, 2011) (Fig. 1A). Similar to the ISC lineage in the PM, gastroblasts (the counterpart of the enteroblast in this region) have been identified and proposed to be the precursor cell that generates these three differentiated cell types (Strand and Micchelli, 2011). Although ISCs in the AM are generally considered to be similar to ISCs in the PM, these two ISC populations have some different properties. For example, proliferation rates and expression of two genes (*Pdp1* and *Stat92E*) are different between AM and PM ISCs (Marianes and Spradling, 2013). Using fluorescence-activated cell sorting (FACS) of ISCs from different regions of the *Drosophila* GI tract, a recent paper has systematically explored gene expression profiles of ISCs in a region-specific manner, and has characterized the roles of some transcription factors, such as GATAe (GATA is a family of transcription factors that are able to bind the DNA sequence ‘GATA’), Snail (Sna; zinc-finger transcriptional repressor) and Ptx1 (paired-type homeobox transcription factor), in global and regional ISC regulation (Dutta et al., 2015b). Future studies are needed to characterize in detail how the function of regional ISCs is regulated by these transcription factors, as well as by other potential molecular factors.

### The mammalian GI tract

In mammals, the structure of GI compartments (including the esophagus, gastric region, small intestine and colon) and the architecture of the epithelia lining these compartments are more complex than those of invertebrates (Fig. 1B). During recent years, specific markers of ISCs have been identified and new *in vivo* and *ex vivo* mouse models for exploring stem cell identity and function have been developed. As a result, our understanding of the regulation of epithelial homeostasis and of regeneration in the mammalian small intestine has improved substantially (Barker, 2014; Clevers, 2013b).

#### The small intestine

The lining of the small intestine is composed of a monostratified epithelium that folds into millions of tubular invaginations known as crypts (Fig. 1B), as well as into numerous finger-like protrusions called villi that project into the intestinal lumen, maximizing surface area for digestion and absorption. The crypts harbor stem cells, Paneth cells and transit amplifying (TA) cells, whereas the villi harbor three main differentiated cell types: ECs, goblet cells and EEs. Paneth cells at the base of the crypt are closely associated with stem cells, and secrete protective antimicrobial substances and the hydrolytic enzyme lysozyme, as well as niche (see Box 1) factors that are crucial for stem cell survival and function. Stem cells within the crypt self-renew and divide to generate TA cells, which in turn proliferate to generate all the functional differentiated cell types of the villi, maintaining tissue homeostasis. In contrast to *Drosophila* enteroblasts, which do not divide but differentiate directly into ECs or EEs, mouse TA cells undergo multiple rounds of cell division to amplify their numbers as they migrate along the crypt axis towards the base of the villus, where they differentiate into various functional cells (Table 1) (Barker et al., 2007; Bjerknes and Cheng, 1999; Hermiston and Gordon, 1995).

The existence of self-renewing and multipotent ISCs in crypts of the small intestine in mice had long been proposed, based on studies of mouse chimeras and the use of mutagen-induced somatic clones (genetically marked cells derived from a single epithelial cell) (Bjerknes and Cheng, 1999; Ponder et al., 1985). However, the recent identification of ISC-specific markers has led to a substantial expansion of ISC studies in mammals (Barker, 2014). Two different stem cell populations have been identified that express different markers and are positioned at different locations in the crypt: crypt base columnar (CBC) stem cells, which sit at the very base of the crypt, and +4 stem cells, which are located four cell diameters apical of the crypt base (see Fig. 1B). CBC stem cells in the adult small intestine express *LGR5* (Leu-rich repeat-containing G protein-coupled receptor 5) (Barker et al., 2007), a Wnt target gene that also labels hair follicle epithelial stem cells, indicating that it might be a general marker for Wnt-activated stem cells (Jaks et al., 2008). Using *Lgr5-lacZ* and *Lgr5-EGFP* reporter mouse models, in which *lacZ* or *EGFP* expression is driven by the endogenous *Lgr5* promoter, Barker et al. found that LGR5 is specifically expressed in CBC cells at the crypt base, and that these LGR5+ CBC cells are evenly distributed between Paneth cells. The ‘stemness’ of LGR5+ CBC cells was confirmed using *in vivo* lineage tracing with an *Lgr5*-driven inducible Cre (see Box 1) recombinase. Marked cells derived from LGR5+ CBC cells quickly grow into clones and expand to form epithelial cell clones that span from the crypt base to the tip of the villus and contain all major epithelial cell types (Barker et al., 2007; Clevers, 2013a).

BMI1, a Polycomb complex protein family member, marks +4 stem cells (Sangiorgi and Capecchi, 2008). The expression pattern of BMI1 in the mouse intestine was first analyzed using RNA *in situ* hybridization, and its function as a marker of stem cells was confirmed using *in vivo* lineage tracing (Sangiorgi and Capecchi, 2008). BMI1+ cells can self-renew, proliferate and generate all the different cell lineages of the small intestine, indicating that BMI1+ cells possess ISC identity. Further support for the function of BMI1+ cells as stem cells was provided by the specific ablation of BMI1+ cells from the crypt, which inhibited epithelial self-renewal and led to crypt loss (Sangiorgi and Capecchi, 2008). Similar to LGR5+ cells, isolated BMI1+ cells can generate epithelial organoids (see Box 1) in culture (Yan et al., 2012). The co-existence but distinct localization of LGR5+ and BMI1+ stem cells suggests that more than one ISC population maintains long-term epithelial homeostasis and regeneration in the mammalian intestine, and that these populations might compensate for each other in certain conditions. Indeed, after the complete ablation of LGR5+ cells, BMI1+ cells can give rise to *Lgr5*-expressing cells and restore intestinal epithelial tissue (Tian et al., 2011). It has been proposed that LGR5+ CBC stem cells are a more active population that is crucial for homeostatic epithelial renewal, whereas BMI1+ ‘+4’ stem cells act as quiescent reserve stem cells that can be activated to restore tissue homeostasis after injury (Barker, 2014). A recent study has provided molecular evidence to support this two-stem-cell model for epithelial regeneration in the mouse small intestine (Li et al., 2014); however, future studies are needed to further characterize the identity of ISC populations and their exact hierarchy.

In humans, epithelial cells from the base of colonic crypts have been purified using an antibody against a surface-expressed WNT target gene, EPH B2 receptor (*EPHB2*). These cells can be expanded *in vitro* and behave as multipotent stem cells (Jung et al., 2011). In a more recent study, *Lgr5-GFP*-positive cells isolated from teratomas (see Box 1) derived from human pluripotent stem cells (hPSCs) generated uniform, long-lived intestinal organoids, thus demonstrating ISC properties (Forster et al., 2014). This further indicates that ISC identity is conserved between mice and humans.

Although regeneration of the small intestine is thus better understood than regeneration of other compartments of the mammalian GI tract, recent studies have made headway in characterizing stem cell populations in the esophagus and stomach, as discussed below.

#### The esophagus

The esophagus, which connects the throat with the stomach, is lined by a stratified epithelium and lacks established niche structures, such as the crypts that are found in the small intestine. The epithelium consists of multiple layers of squamous keratinocytes (see Box 1), with one layer of proliferating basal cells attached to the basement membrane and several layers of differentiated cells that are continuously shed into the lumen and replenished by cells generated at and migrating upward from the basal layer. Although esophageal stem cells are thought to exist in the basal layer, it remains unclear whether all basal cells or only a subpopulation have stem cell identity (Jeong et al., 2015). Studies using nucleotide label retention assays and a combination of cell markers have suggested that a portion of human and mouse esophageal basal cells are self-renewing and are long-lived stem cells (DeWard et al., 2014; Kalabis et al., 2008; Pan et al., 2013). However, pulse-chase experiments in mice using GFP-tagged histone H2B have shown that, although all epithelial cells are labeled after pulse labeling, no label-retaining cells remain in the esophageal epithelium after 4 weeks, indicating that the esophagus does not contain long-lived quiescent stem cells. Furthermore, fate mapping of basal cells using an inducible Cre-*lox* system suggests that all basal cells are functionally equivalent progenitors and that there are no long-lived cycling stem cells (Doupe et al., 2012). Additional studies are needed to clarify these conflicting findings.

#### The stomach

The mammalian stomach has three regions: the forestomach (in mice) or the cardiac region (in humans), the corpus and the pylorus (Fig. 1B). The corpus is the main component of the stomach. Its epithelium is composed of gastric units, crypt-like structures that project deep into the mucosa and can be subdivided into four regions based on distinct cell types. Close to the lumen are mucous cells in the pit region, under which the isthmus harbors fast-dividing stem cells. Below the isthmus is the neck region, which contains gland mucous cells, and at the base are chief cells, which secrete digestive enzymes. Acid-producing parietal cells are scattered throughout all regions. In the pylorus, the crypt-like gland unit is simpler, and in the base, the units contain a population of alkaline mucus-producing cells with few chief or parietal cells (Mills and Shivdasani, 2011).

The first stem cell population to be identified in the stomach, by morphology and labeling using thymidine analogs, maps to a highly proliferative zone of the isthmus (Bjerknes and Cheng, 2002; Lee and Leblond, 1985; Mills and Shivdasani, 2011). Isthmus stem cells can give rise to each of the differentiated cell lineages, although their regulation has not been characterized in detail. It has been proposed that newly generated daughter cells from isthmus stem cells can undergo bidirectional migration: up to the pit to form surface mucus-secreting cells, and down to the base to form zymogenic chief cells through the neck (Mills and Shivdasani, 2011). In addition, these stem cells are thought to form acid-secreting parietal cells and hormone-secreting EEs along the pit-base axis (Fig. 1B). More recently, additional gastric stem cell populations have been identified in mice using Cre-based lineage-tracing methods in different gastric regions (Fig. 1B). Genetic labeling based on *Villin*-promoter–*Cre* (Cre recombinase driven by the *Villin* promoter) has identified a rare stem cell population at varying positions along the gland in the pylorus, which is quiescent in normal conditions and can regenerate all cell types during injury (Qiao et al., 2007). Other studies have identified a stem cell population at the base of the gland in the pylorus using *Lgr5-Cre*-based lineage tracing (Barker et al., 2010b), and a stem cell population in the pylorus and corpus near or under the isthmus region of the gland using *Sox2-Cre* (Arnold et al., 2011). It was further shown that differentiated Troy+ [Troy is encoded by *Tnfrsf19*, and potentially functions as a receptor for lymphotoxin A (Hashimoto et al., 2008)] chief cells, which reside at the bottom of the gastric unit in the corpus, can act as reserve stem cells to regenerate all cell types over a longer period of time (Stange et al., 2013). Together, these studies suggest that the gastric epithelium is highly plastic, and is maintained and regenerated by multiple stem cell populations. The exact lineage relationships between these cells, and how these stem cell populations are coordinated by local niche factors to meet demands during periods of regeneration, remains unclear.

Overall, our understanding of the organization of regenerative processes and of stem cell lineage relationships in the mammalian GI tract has made substantial progress in recent years. The dynamic control of GI stem cell activity during injury, either by infection or by tissue damage, has been explored and characterized in more detail in flies, and we summarize our current understanding of the dynamic regulation of stem cell activity in the following section.

## Signaling pathways that control ISC proliferation and differentiation

**Fig. 2.**
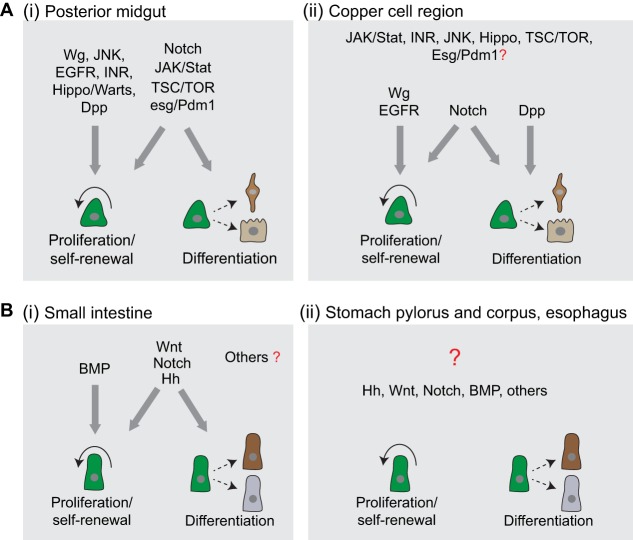
**Stem-cell-regulating signaling pathways in the *Drosophila* and mouse gastrointestinal (GI) tracts.** (A) Regulation of stem cell functions in the *Drosophila* GI tract. (i) In the *Drosophila* posterior midgut (PM), numerous signaling pathways (major pathways depicted) regulate stem cell proliferation, self-renewal and/or differentiation. (ii) In the stomach-like copper cell region (CCR), Wg, EGFR and Notch signaling pathways have similar roles in regulating stem cell functions as the same pathways in the PM, whereas Dpp signaling predominantly regulates stem cell differentiation. The roles of other signaling pathways identified in the CCR have not yet been determined (question mark). (B) Regulation of stem cell functions in the mouse GI tract. (i) In the mouse small intestine, BMP, Wnt, Notch and Hh signaling pathways are required to regulate stem cell functions. However, their roles in regulating stem cell functions in other regions, including the stomach and the esophagus (ii), are less well studied, especially in homeostatic conditions (question mark).

The activity of stem cells along the GI tract needs to be specifically and dynamically regulated to adjust tissue turnover to local and tissue-wide needs. Numerous evolutionarily conserved signaling pathways have been identified to regulate these processes, both in flies and mice. In this section, we summarize our current understanding of the role of these pathways in ISC regulation under homeostatic and regenerative conditions (Fig. 2).

### ISC proliferation and differentiation in *Drosophila*

Signaling pathways that influence ISC proliferation and differentiation in *Drosophila* include Notch (Ohlstein and Spradling, 2007), JAK/Stat (Beebe et al., 2010; Jiang et al., 2009; Lin et al., 2010), Epidermal growth factor receptor (EGFR) (Biteau and Jasper, 2011; Buchon et al., 2010; Jiang and Edgar, 2009; Jiang et al., 2011), Insulin (Choi et al., 2011; O'Brien et al., 2011), Jun-N-terminal kinase (JNK) (Biteau et al., 2008), Wingless (Wg) (Lee et al., 2009; Lin et al., 2008), Target of Rapamycin (TOR) (Amcheslavsky et al., 2011; Kapuria et al., 2012; Quan et al., 2013), Decapentaplegic [Dpp; the *Drosophila* homolog of bone morphogenetic protein (BMP)] (Ayyaz et al., 2015; Guo et al., 2013; Li et al., 2013b; Tian and Jiang, 2014) and Hippo (Karpowicz et al., 2010; Ren et al., 2010; Staley and Irvine, 2010) signaling. ISC differentiation is further controlled by Esg-mediated repression of Pdm1 (Korzelius et al., 2014; Loza-Coll et al., 2014) (Fig. 2A). The combined action of these signaling pathways influences proliferative activity, self-renewal and differentiation in the ISC lineage in response to a wide range of local and systemic cues. (For recent reviews that discuss the detail of ISC regulation by these signaling pathways, see Biteau et al., 2011; Buchon et al., 2013a; Buchon and Osman, 2015; Jiang and Edgar, 2011; Lemaitre and Miguel-Aliaga, 2013.) Recent work has also provided new insights into the integration of these diverse signals. For example, intracellular Ca^2+^ signaling is emerging as a central regulator of ISC proliferation in *Drosophila* in response to a wide range of mitogenic signals (Deng et al., 2015).

The diversity of ISC responses to mitogenic signals along the GI tract remains poorly understood. To date, most studies characterizing the regulation of stem cell function in the *Drosophila* GI tract have focused on the PM. Stem cells in the AM (AM-ISCs), PM (PM-ISCs) and gastric region (GSSCs) share certain properties: all of them are positive for the general ISC marker Esg and can rapidly respond to stress-induced damage by increasing proliferative activity (Biteau et al., 2011; Strand and Micchelli, 2011). PM-ISCs and GSSCs are also regulated in a similar fashion by several signaling pathways, including Wg, EGFR and Notch (Strand and Micchelli, 2011, 2013; Wang et al., 2014) (Fig. 2A). However, their responses to other signaling pathways are diverse. For example, lineage tracing has shown that the loss of Dpp signaling pathway components causes differentiation defects in GSSCs, but not in PM-ISCs (Li et al., 2013a). Sustained Dpp expression along the GI tract is sufficient to induce ectopic copper cell formation in the AM, but not in the PM, indicating that additional regional determinants influence stem cell responses to Dpp signaling. Similarly, the strong activation of JAK/Stat signaling in GSSCs leads to their mis-differentiation, generating ectopic EC-like cells in the copper cell region (Li et al., 2016), whereas, in PM-ISCs, JAK/Stat activation induces proliferation but does not alter differentiation (Jiang et al., 2009) (Fig. 2A). The recent regionally segregated comparison of ISC gene expression profiles (Dutta et al., 2015b) provides a powerful resource that could be used to identify potential factor(s) that specify regional stem cell identity and function in the *Drosophila* GI tract.

### ISC proliferation and differentiation in mammals

In the mammalian small intestine, ISC functions are maintained and regulated by factors from the stem cell niche (see Box 1), which comprises adjacent epithelial cells, pericryptal myofibroblasts, enteric neurons, endothelial cells, intraepithelial lymphocytes and the basement membrane (Walker et al., 2009). As in flies, ISC proliferation and differentiation is controlled by a multitude of signaling pathways, including Wnt, BMP, Hedgehog (Hh) and Notch (Fig. 2B) signaling (Yeung et al., 2011).

A gradient of Wnt activity forms along the crypt axis in the intestinal epithelium of both mice and humans, with the highest activity at the crypt base, decreasing toward the villus (Kosinski et al., 2007; van de Wetering et al., 2002). Wnt signaling is critically important for adult ISC proliferation (Korinek et al., 1998), and loss of Wnt signaling in adult mice, via the genetic deletion of β-catenin (Fevr et al., 2007), the overexpression of the Wnt antagonist Dkk1 (Kuhnert et al., 2004; Pinto et al., 2003) or by the deletion of the Wnt/TCF4 target gene *Myc* (Muncan et al., 2006), leads to complete ablation of intestinal crypts. Wnt signaling further determines cell fates within and the spatial organization of the crypt (Schepers and Clevers, 2012). High Wnt activity in non-proliferating Paneth cells is required for their terminal differentiation (Andreu et al., 2005; van Es et al., 2005a), and this effect is mediated by the Wnt target gene *Sox9* (Bastide et al., 2007; Batlle et al., 2002; Mori-Akiyama et al., 2007).

A gradient of BMP signaling activity also forms along the crypt-villus axis in mouse and human intestines (van der Flier and Clevers, 2009), but this gradient is reversed in relation to Wnt signaling (Haramis et al., 2004; Hardwick et al., 2004; He et al., 2004; Kosinski et al., 2007). Deletion of BMP receptor 1a leads to the expansion of proliferative stem cells and progenitor cells (He et al., 2004), and inhibition of BMP signaling results in the formation of ectopic crypts (Haramis et al., 2004), indicating that BMP signaling negatively regulates ISC proliferation and self-renewal, potentially by inhibiting Wnt signaling (He et al., 2004). These two pathways might also be influenced by Hh signaling, because constitutive activation of Hh signaling by inactivating the receptor patched1 (*Ptch1*) increases BMP signaling and reduces Wnt signaling activity in the adult mouse intestine, resulting in reduced epithelial precursor cells and in the premature differentiation of ECs (van Dop et al., 2009). Accordingly, inhibiting Hh signaling [Sonic hedgehog (*Shh*) and Indian hedgehog (*Ihh*)] results in defective villus formation and in a hyperproliferative epithelium (Madison et al., 2005). In the *Drosophila* hindgut (the posterior part of the GI tract after the midgut), stem cell self-renewal is maintained by Wg signaling, and Hh signaling is required for these cells to exit the cell cycle and differentiate, suggesting that the interaction between Hh and Wg is conserved across organisms (Takashima et al., 2008).

Notch signaling, in turn, seems to have acquired a different function in the mammalian ISC lineage, maintaining the proliferative state of stem and progenitor cells rather than promoting differentiation as it does in flies (van der Flier and Clevers, 2009) (Fig. 2B). The inhibition of Notch signaling in the mouse small intestine leads to cell cycle arrest of proliferative crypt cells and to the rapid conversion of all epithelial cells into secretory goblet cells (Milano et al., 2004; van Es et al., 2005b; Wong et al., 2004). A recent study has shown that blocking Notch signaling using antibodies against Notch receptors in the mouse small intestine leads to the conversion of LGR5+ ISCs into secretory cells. This perturbation is mediated by de-repression of the Wnt signaling pathway, demonstrated by the findings that Notch inhibition leads to activation of Wnt signaling and attenuation of the Wnt pathway rescues phenotypes induced by inhibitory antibodies against Notch (Tian et al., 2015). Ectopic activation of Notch in the mouse small intestine, on the other hand, results in the expansion of proliferative compartments of the crypt, and inhibits the generation of secretory cell types, including goblet cells, EEs and Paneth cells (Fre et al., 2005). In the fly, a recent study suggests that bi-directional Notch signaling plays a role in maintaining ISC fates in asymmetric divisions in the PM that give rise to EEs, suggesting that some aspects of the function of Notch signaling are conserved between flies and vertebrates (Guo and Ohlstein, 2015).

As in the fly, stem cells in different regions of the mammalian GI tract have distinct regional characteristics, allowing them to respond in unique ways to signals to meet local needs. For example, *Lgr5*-expressing stem cells can be found throughout the small intestine and in the pyloric region of the stomach, but not in the main body of the stomach (corpus) (Barker et al., 2010b), nor in the normal esophageal epithelium (von Rahden et al., 2011). This suggests that stem cells along the mammalian GI tract might be differentially regulated by Wnt signaling. In the stomach, stem cells in different regions show a large amount of diversity: Wnt signaling is required to maintain basal stem cells in the distal pyloric region (Fig. 1B) (Barker et al., 2010b), whereas *Lgr5*-negative basal stem cells in the stomach corpus express Troy and exhibit different levels of Wnt activity compared to *Lgr5*-positive pyloric stem cells (Stange et al., 2013) (Fig. 1B). This diversity is further illustrated by the fact that constitutive Notch activation in multipotent progenitors of the corpus significantly increases cell proliferation, leading to adenoma formation, whereas Notch activation in pyloric *Lgr5*-positive stem cells results in normal cell proliferation (Kim and Shivdasani, 2011).

Overall, the regulation of stem cell function in the mammalian stomach and esophagus in homeostatic conditions remains poorly understood. This is partially due to the earlier mentioned debate concerning stem cell identities in the esophagus, but is also due to a lack of stem-cell-specific markers in both the esophagus and stomach. As stem cell markers and stem cell lineages are identified for these gastric regions and explored in detail, we anticipate that the regulation of these cell populations by conserved signaling pathways will become clearer (Fig. 2B).

Although recent studies have provided new information on the regulation of stem cell function by different signaling pathways both in *Drosophila* and mouse GI tracts, several interesting questions remain and warrant further exploration. For example, how are multiple signaling pathways integrated in stem cells to trigger the appropriate response, how do stem cells coordinate symmetric and asymmetric divisions to meet local epithelial needs during regeneration, and how do epithelial environments differentially regulate the plasticity of stem cells and differentiated cells [for example, when does trans-differentiation (see Box 1) occur]? Owing to the simplicity of epithelial structures and the availability of powerful genetic tools, the *Drosophila* GI tract provides an important tool in which to address these questions. It can be anticipated that resolving these open questions will provide important insight not only into physiological regenerative processes, but will also improve our understanding of the origin and progression of proliferative dysfunctions in the GI tract, including the development of cancerous lesions. Our understanding of the development of metaplasias and dysplasias in the GI tract of flies and mammals has greatly advanced in recent years, and is summarized in the following section.

## Metaplasia and dysplasia: understanding molecular mechanisms

Advances in our understanding of the behavior and regulation of GI stem cells under homeostatic conditions have also shed new light on the origin and progression of epithelial disorders in the GI tract. Metaplasia and dysplasia refer to two types of tissue lesions (see Box 1) that are associated with epithelial carcinogenesis (Slack, 2007; Ullman et al., 2009). These lesions are associated with aberrant cell proliferation and differentiation, which eventually leads to loss of tissue homeostasis. A tremendous number of clinical studies have focused on the epidemiology and pathogenesis of intestinal metaplasia and dysplasia in humans (Correa and Houghton, 2007; Harpaz and Polydorides, 2010; Kapoor et al., 2015), and studies in animal models, including in mice and flies, have greatly expanded our knowledge of the mechanisms that cause these lesions at the cellular and molecular level (Li et al., 2013a; Liu et al., 2013; Mari et al., 2014; Quante et al., 2012a). In the closing sections of this Review, we discuss these studies particularly in the context of our emerging understanding of how intrinsic and extrinsic factors cause GI epithelial dysfunction, and how dysregulation of GI stem cells is related to these lesions.

### Metaplasia in the mammalian GI tract

Metaplasias are defined as the replacement of one differentiated cell type by another in a potentially reversible manner. Metaplasias are most likely to occur in epithelial tissues that are frequently exposed to environmental insults and that need to regenerate for tissue repair, such as the airway, the esophagus and the stomach (Slack, 2007). These changes often predispose individuals to the development of cancer (Slack, 2007). Smokers, for example, often exhibit squamous metaplasia in the normally columnar-cell-lined bronchi, and these metaplastic sites are believed to be the site of origin of lung cancers (Auerbach et al., 1961).

**Fig. 3.**
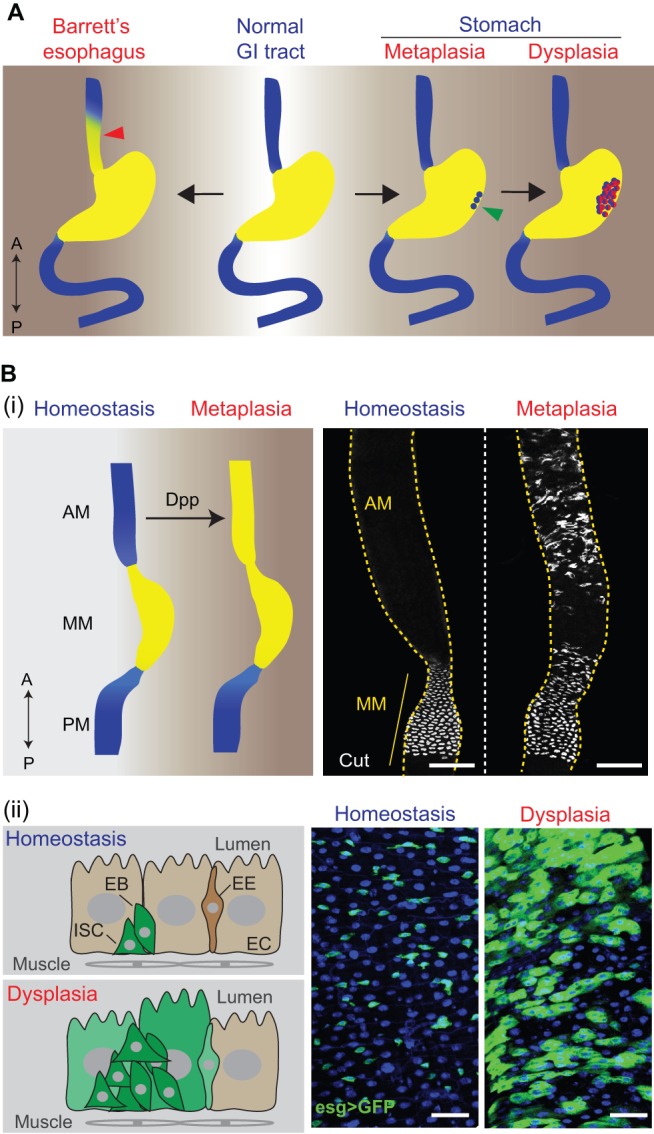
**Metaplasia and dysplasia in the human and *Drosophila* gastrointestinal (GI) tract.** (A) GI pathologies in humans (‘A’, anterior, top; ‘P’, posterior, bottom). Barrett's esophagus (left) is a metaplastic lesion, in which the distal esophageal epithelium (see red arrowhead) is replaced by a stomach- or intestine-like columnar epithelium. Gastric metaplasia (right) is another common metaplastic disease in the human GI tract, in which intestinal epithelial cells (see green arrowhead) are found in the stomach. Gastric metaplasia can develop further into dysplasia (far right, depicted in red), characterized by atypical changes of the epithelial structure due to aberrant cell proliferation and differentiation. (B) GI pathologies in *Drosophila*. (i) Metaplasia in the *Drosophila* anterior midgut (AM) is characterized by ectopic generation of copper cells (CCs), normally restricted to the middle midgut (MM) in homeostatic conditions, in the AM. The schematic on the left summarizes the replacement of AM cells by MM cells (depicted in yellow) during metaplasia. PM, posterior midgut. The images on the right were obtained by immunostaining (using an antibody specific for Cut, a marker for CCs) and confocal microscopy of the midgut region in flies. Dpp overexpression (right-hand panel) generates ectopic acid-producing CCs, indicative of metaplasia (see main text for further details). Scale bars: 100 μm. Microscopy images adapted from Li et al. (2013a). (ii) Dysplasia in the *Drosophila* midgut is characterized by stem cell over-proliferation and abnormal differentiation. The schematic on the left summarizes changes of the epithelial structure during dysplasia. The images on the right were obtained from confocal microscopy of the posterior midgut (blue, DAPI; green, GFP). The stem/progenitor cell marker *esg>GFP* (*esg::Gal4, UAS::GFP*) is only expressed in intestinal stem cells (ISCs) and enteroblasts (EBs) during homeostasis, whereas, in the dysplastic condition, *esg>GFP* begins to be expressed in differentiated cells, including enterocyte (EC)-like cells and enteroendocrine cells (EEs). Scale bars: 25 μm.

Two of the most common metaplasias that affect the mammalian GI tract are Barrett's esophagus (affecting the esophagus) and intestinal metaplasia (occurring in the gastric region) (Fig. 3A). The normal esophagus is lined by multiple layers of squamous cells, which, in Barrett's esophagus, become column-shaped such that the distal esophageal epithelium is eventually replaced by a stomach- or intestine-like columnar epithelium (Falk, 2002). Barrett's esophagus is strongly associated with gastro-esophageal reflux disease (Sarr et al., 1985; Winters et al., 1987), and is the most important risk factor for esophageal adenocarcinoma, especially when dysplastic changes occur in the later stages of the condition (Hvid-Jensen et al., 2011). Current treatment options for Barrett's esophagus include aggressive inhibition of stomach acid production, anti-reflux surgery, chemoprevention and ablation therapy, but there is still no agreement on an optimal treatment or prevention route (Quante et al., 2012a). This is partly due to a lack of knowledge of the exact cellular origins of this condition in humans. Recent studies of mouse models of Barrett's-like metaplasia have implicated several types of cell, including LGR5+ stem cells, which originate in the cardiac region and migrate proximally into the esophagus in response to pro-inflammatory stimuli (Quante et al., 2012b), carbonic anhydrase 4 (CAR4)+/keratin 7 (KRT7)+ residual embryonic cells that expand proximally into the esophagus from their normal postnatal position at the squamocolumnar junction (see Box 1) (Wang et al., 2011), and cells of the submucosal gland ducts that expand and have been shown to generate Barrett's esophagus (Leedham et al., 2008). It is also possible that normal esophageal stem cells within the basal layer change their identity and give rise to Barrett's metaplasia. A better characterization of esophageal stem cells and of cell lineage hierarchy would help to test this hypothesis.

Intestinal metaplasia, another common lesion in the human GI tract, is characterized by the presence of intestinal epithelial cells in the stomach (Correa, 1992). Gastric carcinogenesis often follows a phenotypic path that includes intestinal metaplasia, and that also features gastritis, gastric atrophy, dysplasia and carcinoma (see Box 1). Two stages of metaplasia can be distinguished: complete intestinal metaplasia in the initial phase, where the metaplastic epithelium resembles the mucosa of the small intestine and is lined by absorptive ECs and goblet cells; and incomplete intestinal metaplasia in later stages, where the metaplastic epithelium acquires the morphological features of the large intestine and is lined only by goblet cells (Correa and Houghton, 2007). In both cases, the gastric region starts to express the intestinal marker mucin 2 (MUC2), whereas the gastric marker mucin 6 (MUC6) is lost (Piazuelo et al., 2004). Intestinal metaplasia sometimes continues to develop into dysplasia (see details below) in the gastric region, which can eventually lead to gastric cancer.

Infection by the bacterium *Helicobacter pylori* is the leading cause of gastric cancer, and it is believed that a combination of bacterial virulence factors, environmental insults and the host inflammatory response drive the initiation of gastritis, which can progress to intestinal metaplasia (Correa and Houghton, 2007). Epidemiological studies have reported the beneficial effects of eradicating *H. pylori* for the prevention of gastric cancer development (Correa et al., 2000; Leung et al., 2004; Mera et al., 2005; Uemura et al., 2001). However, the underlying mechanisms of *H. pylori*-driven carcinogenesis and the origins of the metaplastic cells involved remain elusive. Studies in mouse models suggest that *H. pylori*-induced carcinogenesis is associated with chronic inflammation (Wilson and Crabtree, 2007), that infection causes the loss of cells that are important for the appropriate maturation of gastric precursor cells (Li et al., 1996), and that infection increases cell proliferation rates (Cai et al., 2005). Using lineage-tracing approaches, one study has shown that bone-marrow-derived cells can be a source of intestinal metaplasia and gastric cancer (Houghton et al., 2004). How marrow-derived cells are transformed into metaplastic cells remains unclear, and it cannot be ruled out that local gastric stem cells are reprogrammed by the altered immune environment, switch to an intestine-like proliferation/differentiation mode and then generate metaplastic tissue (Correa and Houghton, 2007). More precise characterization of stem cell markers and cell lineages in the gastric region are expected to advance future studies that test this hypothesis.

### Dysplasia in the mammalian GI tract

Dysplasia is characterized by the acquisition of an epithelial structure that has no counterpart in the healthy body (Slack, 2007). During carcinogenesis, dysplasias are found at the neoplastic stage (Rugge et al., 2000), and their rate of progression to invasive carcinomas is very high, both in the gastric region and in the colon (de Vries et al., 2007; Rugge et al., 2000; Ullman et al., 2009). Dysplasias can be classified into low-grade and high-grade dysplasia, depending on the severity of cellular abnormalities (Itzkowitz and Harpaz, 2004). Precancerous metaplastic lesions transition to dysplasias at a relatively slow pace, but the rate of progression can vary, and can be higher in older individuals (Correa et al., 1990; Correa and Houghton, 2007). Furthermore, it is believed that this transition is not a one-way process, because more-advanced lesions can regress to less-advanced lesions (Correa et al., 1990). However, once dysplastic cells cross the basal membrane of the stomach, they become invasive gastric carcinomas, which can be lethal. Dysplasias in the colon are often seen as precursor lesions of colorectal cancer, which accounts for about 10% of all cancers throughout the world (Siegel et al., 2014; Ullman et al., 2009). Proctocolectomy, a surgical technique to remove the rectum and all or part of the colon, is therefore often recommended for cases in which dysplasias are detected in the colon (Itzkowitz and Harpaz, 2004).

Colorectal cancer is strongly associated with inflammatory bowel disease (IBD), including ulcerative colitis and Crohn's disease (Podolsky, 2002). A series of molecular alterations in epithelial cells, including mutations of the Wnt signaling suppressor adenomatosis polyposis coli (*APC*), the tumor suppressor gene *p53*, and the *k-ras* oncogene, drive the development of dysplasia or colorectal cancer from colitis in individuals with IBD (Itzkowitz and Harpaz, 2004; Aust et al., 2002; Brentnall et al., 1994; Burmer et al., 1992; Khor et al., 2011; Neurath, 2014). Inflammatory cells contribute to cancer progression by supplying growth and survival factors that sustain proliferation and limit cell death, and by releasing reactive oxygen species (ROS) that perturb genome maintenance (Grivennikov et al., 2010; Hanahan and Weinberg, 2011). Chronic inflammation, a hallmark of IBD, is thus a critical contributory factor to the molecular changes that drive the progression of colorectal cancer. In addition, recent studies have linked the development of IBD as well as the susceptibility of colitis-associated colorectal cancer to alteration of the intestinal microbiota (Manichanh et al., 2012; Uronis et al., 2009), which could modulate host immune function to favor disease development (Sivan et al., 2015; Vetizou et al., 2015).

Both metaplasia and dysplasia involve the transformation or trans-differentiation of epithelial cells, a process that likely involves changes in the transcriptional programs that establish and maintain cell identities. Indeed, the homeodomain transcription factors *Cdx1* and *Cdx2* have been implicated in changes in GI compartmentalization in mice. During embryonic development, *Cdx1* and *Cdx2* define prospective intestinal cells, but are excluded from prospective stomach regions (Correa, 1992). This expression pattern is maintained in the adult GI tract and is required to maintain the segregation of different compartments (Beck et al., 1999). Intestinal metaplasia in the human stomach is associated with ectopic expression of CDX genes (Silberg et al., 1997), and the forced expression of *Cdx2* using a stomach-specific promoter in mice is sufficient to generate intestinal tissues in the stomach (Mutoh et al., 2002; Silberg et al., 2002). In the mouse intestine, loss of *Cdx2* leads to the formation of a squamous epithelium that resembles the esophagus (Beck et al., 1999), whereas, in Barrett's esophagus, *Cdx2* is ectopically expressed in metaplastic cells (Eda et al., 2003). Furthermore, the overexpression of *Cdx2* and bone morphogenetic protein 4 (*Bmp4*) induces the expression of intestinal genes in the mouse esophagus (Mari et al., 2014), whereas activation of two transcription factors, *Sox2* and *Stat3*, transforms basal progenitor cells into malignant cells, causing squamous cancer (Liu et al., 2013). These studies suggest that deregulation of crucial transcription factors or growth factor pathways triggers a cascade of events that eventually lead to metaplasia and dysplasia.

The development of metaplasias and dysplasias is tightly linked to deregulation of GI stem cell function, and gaining a deeper understanding of the regulation of stem cell activity, identity and maintenance is crucial if we are to understand the origin and progression of these pathologies. Work in the *Drosophila* GI tract has made substantial progress towards this goal, as highlighted below.

### Metaplasia and dysplasia in the *Drosophila* GI tract

The adult *Drosophila* GI tract is emerging as a powerful model in which to explore in mechanistic detail the origin and progression of metaplasias and dysplasias as a consequence of dysfunctions in ISC biology, microbe-host interactions and epithelial deregulation (Biteau et al., 2011; Lemaitre and Miguel-Aliaga, 2013). During aging or after a pathogenic infection, compartmentalization of the GI tract is disturbed, as revealed by a strong alteration in gene expression patterns in these two conditions (Buchon et al., 2013b). However, signaling mechanisms that maintain compartment identities are only beginning to be understood. Dpp signaling activity forms a gradient near the boundary between the MM and the PM. This gradient seems to segregate stem cell identities in those two regions (Guo et al., 2013; Li et al., 2013a). Ectopic and chronic activation of Dpp signaling in the AM can lead to the aberrant development of copper cells, which are normally restricted to the MM, in this region; a metaplasia similar to Barrett's esophagus in humans (Li et al., 2013a) (Fig. 3B). Interestingly, this metaplasia is only observed when Dpp is overexpressed in ECs using a strong EC driver (Li et al., 2013a), and not when it is expressed using a visceral muscle driver (Driver and Ohlstein, 2014), indicating that the source and/or strength of the Dpp signal determines the response of AM cells. Metaplasia induced by Dpp overexpression in the AM is mediated by the downstream transcription factor Labial (Li et al., 2013a), which also plays an important role in GI compartmentalization in the larval stage, specifically for MM development (Nakagoshi, 2005), similar to the role of CDX genes in mammals. Metaplasias thus seem to be driven by the ectopic activation of developmental pathways in the adult. This metaplasia is not seen in the PM, supporting the notion that ISCs in different regions of the GI tract possess distinct characteristics. It is possible that ‘pan-ISC’ transcription factors along the GI tract determine a general stem cell identity, and region-specific transcription factors further refine stem cell properties. The analysis of regional ISC gene expression profiles and of regional properties of ISCs by lineage tracing supports this notion (Dutta et al., 2015a; Marianes and Spradling, 2013).

Dysplasia is a common age-related dysfunction of the *Drosophila* GI tract (Fig. 3B). In aging flies, ISCs become hyper-proliferative, leading to the accumulation of mis-differentiated cells that co-express stem and progenitor cell markers (such as Dl and Esg), and differentiation markers (such as Notch signaling activity and polyploidy) (Biteau et al., 2008; Buchon et al., 2009; Hochmuth et al., 2011). These dysplastic changes can result in epithelial barrier dysfunction, which is strongly associated with fly death (Rera et al., 2012). Age-related intestinal dysplasia is caused by increased JNK and/or Platelet-derived growth factor (PDGF)/Vascular endothelial growth factor (VEGF) signaling activity in the aging intestine (Biteau et al., 2008; Choi et al., 2008). In response to oxidative stress or pathogenic infection, JNK signaling and JNK-mediated cytokine/JAK/Stat signaling can also be chronically activated in *Drosophila*, leading to similar dysplastic phenotypes (Biteau et al., 2008; Buchon et al., 2009; Hochmuth et al., 2011). In addition, a recent study shows that dysregulation of ISC niche signals, including EGFR ligands and cytokines that activate JAK/Stat signaling, contribute to the development of dysplasia and tumorigenesis from Notch-defective ISCs (Patel et al., 2015). Furthermore, defects in endocytic degradation can cause intestinal dysplasia (Nagy et al., 2016).

A number of factors that contribute to dysplasia in the aging intestine, including a decline of mitochondrial function in stem and progenitor cells, dysbiosis of gut commensals, inflammatory signals from the fat body, increased endoplasmic reticulum (ER) stress and frequent somatic mutation in ISCs in the intestinal epithelium, have now been identified (Chen et al., 2014; Clark et al., 2015; Guo et al., 2014; Rera et al., 2011; Siudeja et al., 2015; Wang et al., 2015b). Limiting age-related dysplastic changes by genetically manipulating ISCs to avoid hyper-proliferation is sufficient to extend *Drosophila* lifespan (Biteau et al., 2010). Understanding the underlying mechanisms that contribute to age-related dysplasia is thus likely to help identify interventions that not only improve intestinal function, but also benefit the health of the whole organism. Several interventions that specifically target these factors have been shown to extend the lifespan of flies reared under laboratory conditions (Ayyaz and Jasper, 2013; Chen et al., 2014; Clark et al., 2015; Guo et al., 2014; Rera et al., 2011; Wang et al., 2015b).

## Conclusions and perspectives

It is clear that a large number of parallels can be drawn between stem cell function and regulation, as well as the control of regenerative processes, in the GI tract of flies and mammals. The rich mechanistic insight provided by the fly system is thus expected to complement studies made in mice to enhance our understanding of stem cell function in the GI tract of humans. Moreover, the evolutionary conservation of regenerative processes in different regions of invertebrate and vertebrate GI tracts indicate that current research on the origin of pathological dysfunctions in *Drosophila* GI epithelia will generate important insight into human pathologies. *Drosophila* research benefits from the short lifespan, relative genetic, morphological and functional simplicity, and experimental accessibility of the model. Crucially, the sophisticated genetic tools available for spatiotemporally controlled genetic perturbations and for selective lineage tracing enable rigorous characterization of the genetic control of regeneration in both homeostatic and pathological conditions. Given the advances made so far, we anticipate that studies using *Drosophila* will soon provide insight that will inform the development of targeted interventions to prevent or treat human pathologies of the GI tract.

## References

[DMM024232C1] Amcheslavsky A., Ito N., Jiang J. and Ip Y. T. (2011). Tuberous sclerosis complex and Myc coordinate the growth and division of Drosophila intestinal stem cells. *J. Cell Biol.* 193, 695-710. 10.1083/jcb.201103018 21555458 PMC3166862

[DMM024232C2] Andreu P., Colnot S., Godard C., Gad S., Chafey P., Niwa-Kawakita M., Laurent-Puig P., Kahn A., Robine S., Perret C. et al. (2005). Crypt-restricted proliferation and commitment to the Paneth cell lineage following Apc loss in the mouse intestine. *Development* 132, 1443-1451. 10.1242/dev.01700 15716339

[DMM024232C3] Arnold K., Sarkar A., Yram M. A., Polo J. M., Bronson R., Sengupta S., Seandel M., Geijsen N. and Hochedlinger K. (2011). Sox2(+) adult stem and progenitor cells are important for tissue regeneration and survival of mice. *Cell Stem Cell* 9, 317-329. 10.1016/j.stem.2011.09.001 21982232 PMC3538360

[DMM024232C4] Auerbach O., Stout A. P., Hammond E. C. and Garfinkel L. (1961). Changes in bronchial epithelium in relation to cigarette smoking and in relation to lung cancer. *N. Engl. J. Med.* 265, 253-267. 10.1056/NEJM196108102650601 13685078

[DMM024232C5] Aust D. E., Terdiman J. P., Willenbucher R. F., Chang C. G., Molinaro-Clark A., Baretton G. B., Loehrs U. and Waldman F. M. (2002). The APC/beta-catenin pathway in ulcerative colitis-related colorectal carcinomas: a mutational analysis. *Cancer* 94, 1421-1427. 10.1002/cncr.10334 11920497

[DMM024232C6] Ayyaz A. and Jasper H. (2013). Intestinal inflammation and stem cell homeostasis in aging Drosophila melanogaster. *Front. Cell. Infect. Microbiol.* 3, 98 10.3389/fcimb.2013.00098 24380076 PMC3863754

[DMM024232C7] Ayyaz A., Li H. and Jasper H. (2015). Haemocytes control stem cell activity in the Drosophila intestine. *Nat. Cell Biol.* 17, 736-748. 10.1038/ncb3174 26005834 PMC4449816

[DMM024232C8] Barker N. (2014). Adult intestinal stem cells: critical drivers of epithelial homeostasis and regeneration. *Nat. Rev. Mol. Cell Biol.* 15, 19-33. 10.1038/nrm3721 24326621

[DMM024232C9] Barker N., van Es J. H., Kuipers J., Kujala P., van den Born M., Cozijnsen M., Haegebarth A., Korving J., Begthel H., Peters P. J. et al. (2007). Identification of stem cells in small intestine and colon by marker gene Lgr5. *Nature* 449, 1003-1007. 10.1038/nature06196 17934449

[DMM024232C10] Barker N., Bartfeld S. and Clevers H. (2010a). Tissue-resident adult stem cell populations of rapidly self-renewing organs. *Cell Stem Cell* 7, 656-670. 10.1016/j.stem.2010.11.016 21112561

[DMM024232C11] Barker N., Huch M., Kujala P., van de Wetering M., Snippert H. J., van Es J. H., Sato T., Stange D. E., Begthel H., van den Born M. et al. (2010b). Lgr5(+ve) stem cells drive self-renewal in the stomach and build long-lived gastric units in vitro. *Cell Stem Cell* 6, 25-36. 10.1016/j.stem.2009.11.013 20085740

[DMM024232C12] Bastide P., Darido C., Pannequin J., Kist R., Robine S., Marty-Double C., Bibeau F., Scherer G., Joubert D., Hollande F. et al. (2007). Sox9 regulates cell proliferation and is required for Paneth cell differentiation in the intestinal epithelium. *J. Cell Biol.* 178, 635-648. 10.1083/jcb.200704152 17698607 PMC2064470

[DMM024232C13] Batlle E., Henderson J. T., Beghtel H., van den Born M. M. W., Sancho E., Huls G., Meeldijk J., Robertson J., van de Wetering M., Pawson T. et al. (2002). Beta-catenin and TCF mediate cell positioning in the intestinal epithelium by controlling the expression of EphB/ephrinB. *Cell* 111, 251-263. 10.1016/S0092-8674(02)01015-2 12408869

[DMM024232C14] Beck F., Chawengsaksophak K., Waring P., Playford R. J. and Furness J. B. (1999). Reprogramming of intestinal differentiation and intercalary regeneration in Cdx2 mutant mice. *Proc. Natl. Acad. Sci. USA* 96, 7318-7323. 10.1073/pnas.96.13.7318 10377412 PMC22083

[DMM024232C15] Beebe K., Lee W.-C. and Micchelli C. A. (2010). JAK/STAT signaling coordinates stem cell proliferation and multilineage differentiation in the Drosophila intestinal stem cell lineage. *Dev. Biol.* 338, 28-37. 10.1016/j.ydbio.2009.10.045 19896937

[DMM024232C16] Beehler-Evans R. and Micchelli C. A. (2015). Generation of enteroendocrine cell diversity in midgut stem cell lineages. *Development* 142, 654-664. 10.1242/dev.114959 25670792 PMC4325375

[DMM024232C17] Biteau B. and Jasper H. (2011). EGF signaling regulates the proliferation of intestinal stem cells in Drosophila. *Development* 138, 1045-1055. 10.1242/dev.056671 21307097 PMC3042864

[DMM024232C18] Biteau B. and Jasper H. (2014). Slit/Robo signaling regulates cell fate decisions in the intestinal stem cell lineage of Drosophila. *Cell Rep.* 7, 1867-1875. 10.1016/j.celrep.2014.05.024 24931602 PMC4086754

[DMM024232C19] Biteau B., Hochmuth C. E. and Jasper H. (2008). JNK activity in somatic stem cells causes loss of tissue homeostasis in the aging Drosophila gut. *Cell Stem Cell* 3, 442-455. 10.1016/j.stem.2008.07.024 18940735 PMC3225008

[DMM024232C20] Biteau B., Karpac J., Supoyo S., DeGennaro M., Lehmann R. and Jasper H. (2010). Lifespan extension by preserving proliferative homeostasis in Drosophila. *PLoS Genet.* 6, e1001159 10.1371/journal.pgen.1001159 20976250 PMC2954830

[DMM024232C21] Biteau B., Hochmuth C. E. and Jasper H. (2011). Maintaining tissue homeostasis: dynamic control of somatic stem cell activity. *Cell Stem Cell* 9, 402-411. 10.1016/j.stem.2011.10.004 22056138 PMC3212030

[DMM024232C22] Bjerknes M. and Cheng H. (1999). Clonal analysis of mouse intestinal epithelial progenitors. *Gastroenterology* 116, 7-14. 10.1016/S0016-5085(99)70222-2 9869596

[DMM024232C23] Bjerknes M. and Cheng H. (2002). Multipotential stem cells in adult mouse gastric epithelium. *Am. J. Physiol. Gastrointest. Liver Physiol.* 283, G767-G777. 10.1152/ajpgi.00415.2001 12181193

[DMM024232C24] Brentnall T. A., Crispin D. A., Rabinovitch P. S., Haggitt R. C., Rubin C. E., Stevens A. C. and Burmer G. C. (1994). Mutations in the p53 gene: an early marker of neoplastic progression in ulcerative colitis. *Gastroenterology* 107, 369-378. 8039614 10.1016/0016-5085(94)90161-9

[DMM024232C25] Buchon N. and Osman D. (2015). All for one and one for all: regionalization of the Drosophila intestine. *Insect Biochem. Mol. Biol.* 67, 2-8. 10.1016/j.ibmb.2015.05.015 26044368

[DMM024232C26] Buchon N., Broderick N. A., Chakrabarti S. and Lemaitre B. (2009). Invasive and indigenous microbiota impact intestinal stem cell activity through multiple pathways in Drosophila. *Genes Dev.* 23, 2333-2344. 10.1101/gad.1827009 19797770 PMC2758745

[DMM024232C27] Buchon N., Broderick N. A., Kuraishi T. and Lemaitre B. (2010). Drosophila EGFR pathway coordinates stem cell proliferation and gut remodeling following infection. *BMC Biol.* 8, 152 10.1186/1741-7007-8-152 21176204 PMC3022776

[DMM024232C28] Buchon N., Broderick N. A. and Lemaitre B. (2013a). Gut homeostasis in a microbial world: insights from Drosophila melanogaster. *Nat. Rev. Microbiol.* 11, 615-626. 10.1038/nrmicro3074 23893105

[DMM024232C29] Buchon N., Osman D., David F. P. A., Fang H. Y., Boquete J.-P., Deplancke B. and Lemaitre B. (2013b). Morphological and molecular characterization of adult midgut compartmentalization in Drosophila. *Cell Rep.* 3, 1725-1738. 10.1016/j.celrep.2013.04.001 23643535

[DMM024232C30] Burmer G. C., Rabinovitch P. S., Haggitt R. C., Crispin D. A., Brentnall T. A., Kolli V. R., Stevens A. C. and Rubin C. E. (1992). Neoplastic progression in ulcerative colitis: histology, DNA content, and loss of a p53 allele. *Gastroenterology* 103, 1602-1610. 1358743 10.1016/0016-5085(92)91184-6

[DMM024232C31] Cai X., Carlson J., Stoicov C., Li H., Wang T. C. and Houghton J. (2005). Helicobacter felis eradication restores normal architecture and inhibits gastric cancer progression in C57BL/6 mice. *Gastroenterology* 128, 1937-1952. 10.1053/j.gastro.2005.02.066 15940628

[DMM024232C32] Chen H., Zheng X. and Zheng Y. (2014). Age-associated loss of lamin-B leads to systemic inflammation and gut hyperplasia. *Cell* 159, 829-843. 10.1016/j.cell.2014.10.028 25417159 PMC4243052

[DMM024232C33] Choi N.-H., Kim J.-G., Yang D.-J., Kim Y.-S. and Yoo M.-A. (2008). Age-related changes in Drosophila midgut are associated with PVF2, a PDGF/VEGF-like growth factor. *Aging Cell* 7, 318-334. 10.1111/j.1474-9726.2008.00380.x 18284659 PMC2408640

[DMM024232C34] Choi N. H., Lucchetta E. and Ohlstein B. (2011). Nonautonomous regulation of Drosophila midgut stem cell proliferation by the insulin-signaling pathway. *Proc. Natl. Acad. Sci. USA* 108, 18702-18707. 10.1073/pnas.1109348108 22049341 PMC3219098

[DMM024232C35] Clark R. I., Salazar A., Yamada R., Fitz-Gibbon S., Morselli M., Alcaraz J., Rana A., Rera M., Pellegrini M., Ja W. W. et al. (2015). Distinct shifts in microbiota composition during Drosophila aging impair intestinal function and drive mortality. *Cell Rep.* 12, 1656-1667. 10.1016/j.celrep.2015.08.004 26321641 PMC4565751

[DMM024232C36] Clevers H. (2013a). A gutsy approach to stem cells and signalling: an interview with Hans Clevers. *Dis. Model. Mech.* 6, 1053-1056. 10.1242/dmm.013367 24046385 PMC3759325

[DMM024232C37] Clevers H. (2013b). The intestinal crypt, a prototype stem cell compartment. *Cell* 154, 274-284. 10.1016/j.cell.2013.07.004 23870119

[DMM024232C38] Correa P. (1992). Human gastric carcinogenesis: a multistep and multifactorial process--First American Cancer Society Award Lecture on Cancer Epidemiology and Prevention. *Cancer Res.* 52, 6735-6740. 1458460

[DMM024232C39] Correa P. and Houghton J. (2007). Carcinogenesis of Helicobacter pylori. *Gastroenterology* 133, 659-672. 10.1053/j.gastro.2007.06.026 17681184

[DMM024232C40] Correa P., Haenszel W., Cuello C., Zavala D., Fontham E., Zarama G., Tannenbaum S., Collazos T. and Ruiz B. (1990). Gastric precancerous process in a high risk population: cohort follow-up. *Cancer Res.* 50, 4737-4740. 2369748

[DMM024232C41] Correa P., Fontham E. T. H., Bravo J. C., Bravo L. E., Ruiz B., Zarama G., Realpe J. L., Malcom G. T., Li D., Johnson W. D. et al. (2000). Chemoprevention of gastric dysplasia: randomized trial of antioxidant supplements and anti-helicobacter pylori therapy. *J. Natl. Cancer Inst.* 92, 1881-1888. 10.1093/jnci/92.23.1881 11106679

[DMM024232C42] de Vries A. C., Haringsma J. and Kuipers E. J. (2007). The detection, surveillance and treatment of premalignant gastric lesions related to Helicobacter pylori infection. *Helicobacter* 12, 1-15. 10.1111/j.1523-5378.2007.00475.x 17241295

[DMM024232C43] Deng H., Gerencser A. A. and Jasper H. (2015). Signal integration by Ca(2+) regulates intestinal stem-cell activity. *Nature* 528, 212-217. 10.1038/nature16170 26633624 PMC4669953

[DMM024232C44] DeWard A. D., Cramer J. and Lagasse E. (2014). Cellular heterogeneity in the mouse esophagus implicates the presence of a nonquiescent epithelial stem cell population. *Cell Rep.* 9, 701-711. 10.1016/j.celrep.2014.09.027 25373907 PMC4223874

[DMM024232C45] Doupe D. P., Alcolea M. P., Roshan A., Zhang G., Klein A. M., Simons B. D. and Jones P. H. (2012). A single progenitor population switches behavior to maintain and repair esophageal epithelium. *Science* 337, 1091-1093. 10.1126/science.1218835 22821983 PMC3527005

[DMM024232C46] Driver I. and Ohlstein B. (2014). Specification of regional intestinal stem cell identity during Drosophila metamorphosis. *Development* 141, 1848-1856. 10.1242/dev.104018 24700821 PMC3994771

[DMM024232C47] Dutta D., Buchon N., Xiang J. and Edgar B. A. (2015a). Regional Cell Specific RNA Expression Profiling of FACS Isolated Drosophila Intestinal Cell Populations. *Curr. Protoc. Stem Cell Biol.* 34, 2F.2.1-2F.2.14. 10.1002/9780470151808.sc02f02s34 26237570

[DMM024232C48] Dutta D., Dobson A. J., Houtz P. L., Glasser C., Revah J., Korzelius J., Patel P. H., Edgar B. A. and Buchon N. (2015b). Regional cell-specific transcriptome mapping reveals regulatory complexity in the adult Drosophila midgut. *Cell Rep.* 12, 346-358. 10.1016/j.celrep.2015.06.009 26146076

[DMM024232C49] Eda A., Osawa H., Satoh K., Yanaka I., Kihira K., Ishino Y., Mutoh H. and Sugano K. (2003). Aberrant expression of CDX2 in Barrett's epithelium and inflammatory esophageal mucosa. *J. Gastroenterol.* 38, 14-22. 10.1007/s005350300001 12560917

[DMM024232C50] Falk G. W. (2002). Barrett's esophagus. *Gastroenterology* 122, 1569-1591. 10.1053/gast.2002.33427 12016424

[DMM024232C51] Fevr T., Robine S., Louvard D. and Huelsken J. (2007). Wnt/beta-catenin is essential for intestinal homeostasis and maintenance of intestinal stem cells. *Mol. Cell. Biol.* 27, 7551-7559. 10.1128/MCB.01034-07 17785439 PMC2169070

[DMM024232C52] Forster R., Chiba K., Schaeffer L., Regalado S. G., Lai C. S., Gao Q., Kiani S., Farin H. F., Clevers H., Cost G. J. et al. (2014). Human intestinal tissue with adult stem cell properties derived from pluripotent stem cells. *Stem Cell Rep.* 2, 838-852. 10.1016/j.stemcr.2014.05.001 PMC4050346 24936470

[DMM024232C53] Fre S., Huyghe M., Mourikis P., Robine S., Louvard D. and Artavanis-Tsakonas S. (2005). Notch signals control the fate of immature progenitor cells in the intestine. *Nature* 435, 964-968. 10.1038/nature03589 15959516

[DMM024232C54] Grivennikov S. I., Greten F. R. and Karin M. (2010). Immunity, inflammation, and cancer. *Cell* 140, 883-899. 10.1016/j.cell.2010.01.025 20303878 PMC2866629

[DMM024232C55] Guo Z. and Ohlstein B. (2015). Stem cell regulation. Bidirectional Notch signaling regulates Drosophila intestinal stem cell multipotency. *Science* 350, aab0988 10.1126/science.aab0988 26586765 PMC5431284

[DMM024232C56] Guo Z., Driver I. and Ohlstein B. (2013). Injury-induced BMP signaling negatively regulates Drosophila midgut homeostasis. *J. Cell Biol.* 201, 945-961. 10.1083/jcb.201302049 23733344 PMC3678160

[DMM024232C57] Guo L., Karpac J., Tran S. L. and Jasper H. (2014). PGRP-SC2 promotes gut immune homeostasis to limit commensal dysbiosis and extend lifespan. *Cell* 156, 109-122. 10.1016/j.cell.2013.12.018 24439372 PMC3928474

[DMM024232C58] Hanahan D. and Weinberg R. A. (2011). Hallmarks of cancer: the next generation. *Cell* 144, 646-674. 10.1016/j.cell.2011.02.013 21376230

[DMM024232C59] Haramis A.-P. G., Begthel H., van den Born M., van Es J., Jonkheer S., Offerhaus G. J. A. and Clevers H. (2004). De novo crypt formation and juvenile polyposis on BMP inhibition in mouse intestine. *Science* 303, 1684-1686. 10.1126/science.1093587 15017003

[DMM024232C60] Hardwick J. C. H., Van Den Brink G. R., Bleuming S. A., Ballester I., Van Den Brande J. M. H., Keller J. J., Offerhaus G. J. A., Van Deventer S. J. H. and Peppelenbosch M. P. (2004). Bone morphogenetic protein 2 is expressed by, and acts upon, mature epithelial cells in the colon. *Gastroenterology* 126, 111-121. 10.1053/j.gastro.2003.10.067 14699493

[DMM024232C61] Harpaz N. and Polydorides A. D. (2010). Colorectal dysplasia in chronic inflammatory bowel disease: pathology, clinical implications, and pathogenesis. *Arch. Pathol. Lab. Med.* 134, 876-895. 20524866 10.5858/134.6.876

[DMM024232C62] Hashimoto T., Schlessinger D. and Cui C.-Y. (2008). Troy binding to lymphotoxin-alpha activates NF kappa B mediated transcription. *Cell Cycle* 7, 106-111. 10.4161/cc.7.1.5135 18202551

[DMM024232C63] He X. C., Zhang J., Tong W.-G., Tawfik O., Ross J., Scoville D. H., Tian Q., Zeng X., He X., Wiedemann L. M. et al. (2004). BMP signaling inhibits intestinal stem cell self-renewal through suppression of Wnt-beta-catenin signaling. *Nat. Genet.* 36, 1117-1121. 10.1038/ng1430 15378062

[DMM024232C64] Hermiston M. L. and Gordon J. I. (1995). Organization of the crypt-villus axis and evolution of its stem cell hierarchy during intestinal development. *Am. J. Physiol.* 268, G813-G822. 7762665 10.1152/ajpgi.1995.268.5.G813

[DMM024232C65] Hochmuth C. E., Biteau B., Bohmann D. and Jasper H. (2011). Redox regulation by Keap1 and Nrf2 controls intestinal stem cell proliferation in Drosophila. *Cell Stem Cell* 8, 188-199. 10.1016/j.stem.2010.12.006 21295275 PMC3035938

[DMM024232C66] Houghton J., Stoicov C., Nomura S., Rogers A. B., Carlson J., Li H., Cai X., Fox J. G., Goldenring J. R. and Wang T. C. (2004). Gastric cancer originating from bone marrow-derived cells. *Science* 306, 1568-1571. 10.1126/science.1099513 15567866

[DMM024232C67] Hvid-Jensen F., Pedersen L., Drewes A. M., Sorensen H. T. and Funch-Jensen P. (2011). Incidence of adenocarcinoma among patients with Barrett's esophagus. *N. Engl. J. Med.* 365, 1375-1383. 10.1056/NEJMoa1103042 21995385

[DMM024232C68] Itzkowitz S. H. and Harpaz N. (2004). Diagnosis and management of dysplasia in patients with inflammatory bowel diseases. *Gastroenterology* 126, 1634-1648. 10.1053/j.gastro.2004.03.025 15168373

[DMM024232C69] Jaks V., Barker N., Kasper M., van Es J. H., Snippert H. J., Clevers H. and Toftgård R. (2008). Lgr5 marks cycling, yet long-lived, hair follicle stem cells. *Nat. Genet.* 40, 1291-1299. 10.1038/ng.239 18849992

[DMM024232C70] Jeong Y., Rhee H., Martin S., Klass D., Lin Y., Nguyen L. X. T., Feng W. and Diehn M. (2015). Identification and genetic manipulation of human and mouse oesophageal stem cells. *Gut* 309, G216-G228. 10.1136/gutjnl-2014-308491 25897018

[DMM024232C71] Jiang H. and Edgar B. A. (2009). EGFR signaling regulates the proliferation of Drosophila adult midgut progenitors. *Development* 136, 483-493. 10.1242/dev.026955 19141677 PMC2687592

[DMM024232C72] Jiang H. and Edgar B. A. (2011). Intestinal stem cells in the adult Drosophila midgut. *Exp. Cell Res.* 317, 2780-2788. 10.1016/j.yexcr.2011.07.020 21856297 PMC6141237

[DMM024232C73] Jiang H., Patel P. H., Kohlmaier A., Grenley M. O., McEwen D. G. and Edgar B. A. (2009). Cytokine/Jak/Stat signaling mediates regeneration and homeostasis in the Drosophila midgut. *Cell* 137, 1343-1355. 10.1016/j.cell.2009.05.014 19563763 PMC2753793

[DMM024232C74] Jiang H., Grenley M. O., Bravo M.-J., Blumhagen R. Z. and Edgar B. A. (2011). EGFR/Ras/MAPK signaling mediates adult midgut epithelial homeostasis and regeneration in Drosophila. *Cell Stem Cell* 8, 84-95. 10.1016/j.stem.2010.11.026 21167805 PMC3021119

[DMM024232C75] Jung P., Sato T., Merlos-Suárez A., Barriga F. M., Iglesias M., Rossell D., Auer H., Gallardo M., Blasco M. A., Sancho E. et al. (2011). Isolation and in vitro expansion of human colonic stem cells. *Nat. Med.* 17, 1225-1227. 10.1038/nm.2470 21892181

[DMM024232C76] Kalabis J., Oyama K., Okawa T., Nakagawa H., Michaylira C. Z., Stairs D. B., Figueiredo J.-L., Mahmood U., Diehl J. A., Herlyn M. et al. (2008). A subpopulation of mouse esophageal basal cells has properties of stem cells with the capacity for self-renewal and lineage specification. *J. Clin. Invest.* 118, 3860-3869. 10.1172/jci35012 19033657 PMC2579884

[DMM024232C77] Kapoor H., Agrawal D. K. and Mittal S. K. (2015). Barrett's esophagus: recent insights into pathogenesis and cellular ontogeny. *Transl. Res.* 166, 28-40. 10.1016/j.trsl.2015.01.009 25701368

[DMM024232C78] Kapuria S., Karpac J., Biteau B., Hwangbo D. and Jasper H. (2012). Notch-mediated suppression of TSC2 expression regulates cell differentiation in the Drosophila intestinal stem cell lineage. *PLoS Genet.* 8, e1003045 10.1371/journal.pgen.1003045 23144631 PMC3493453

[DMM024232C79] Karpowicz P., Perez J. and Perrimon N. (2010). The Hippo tumor suppressor pathway regulates intestinal stem cell regeneration. *Development* 137, 4135-4145. 10.1242/dev.060483 21098564 PMC2990205

[DMM024232C80] Khor B., Gardet A. and Xavier R. J. (2011). Genetics and pathogenesis of inflammatory bowel disease. *Nature* 474, 307-317. 10.1038/nature10209 21677747 PMC3204665

[DMM024232C81] Kim T.-H. and Shivdasani R. A. (2011). Notch signaling in stomach epithelial stem cell homeostasis. *J. Exp. Med.* 208, 677-688. 10.1084/jem.20101737 21402740 PMC3137787

[DMM024232C82] Korinek V., Barker N., Moerer P., van Donselaar E., Huls G., Peters P. J. and Clevers H. (1998). Depletion of epithelial stem-cell compartments in the small intestine of mice lacking Tcf-4. *Nat. Genet.* 19, 379-383. 10.1038/1270 9697701

[DMM024232C83] Korzelius J., Naumann S. K., Loza-Coll M. A., Chan J. S., Dutta D., Oberheim J., Glasser C., Southall T. D., Brand A. H., Jones D. L. et al. (2014). Escargot maintains stemness and suppresses differentiation in Drosophila intestinal stem cells. *EMBO J.* 33, 2967-2982. 10.15252/embj.201489072 25298397 PMC4282643

[DMM024232C84] Kosinski C., Li V. S. W., Chan A. S. Y., Zhang J., Ho C., Tsui W. Y., Chan T. L., Mifflin R. C., Powell D. W., Yuen S. T. et al. (2007). Gene expression patterns of human colon tops and basal crypts and BMP antagonists as intestinal stem cell niche factors. *Proc. Natl. Acad. Sci. USA* 104, 15418-15423. 10.1073/pnas.0707210104 17881565 PMC2000506

[DMM024232C85] Kuhnert F., Davis C. R., Wang H.-T., Chu P., Lee M., Yuan J., Nusse R. and Kuo C. J. (2004). Essential requirement for Wnt signaling in proliferation of adult small intestine and colon revealed by adenoviral expression of Dickkopf-1. *Proc. Natl. Acad. Sci. USA* 101, 266-271. 10.1073/pnas.2536800100 14695885 PMC314174

[DMM024232C86] Lee E. R. and Leblond C. P. (1985). Dynamic histology of the antral epithelium in the mouse stomach: II. Ultrastructure and renewal of isthmal cells. *Am. J. Anat.* 172, 205-224. 10.1002/aja.1001720304 3993597

[DMM024232C87] Lee W.-C., Beebe K., Sudmeier L. and Micchelli C. A. (2009). Adenomatous polyposis coli regulates Drosophila intestinal stem cell proliferation. *Development* 136, 2255-2264. 10.1242/dev.035196 19502486 PMC13471719

[DMM024232C88] Leedham S. J., Preston S. L., McDonald S. A. C., Elia G., Bhandari P., Poller D., Harrison R., Novelli M. R., Jankowski J. A. and Wright N. A. (2008). Individual crypt genetic heterogeneity and the origin of metaplastic glandular epithelium in human Barrett's oesophagus. *Gut* 57, 1041-1048. 10.1136/gut.2007.143339 18305067 PMC2564832

[DMM024232C89] Lemaitre B. and Miguel-Aliaga I. (2013). The digestive tract of Drosophila melanogaster. *Annu. Rev. Genet.* 47, 377-404. 10.1146/annurev-genet-111212-133343 24016187

[DMM024232C90] Leung W. K., Lin S.-R., Ching J. Y. L., To K.-F., Ng E. K. W., Chan F. K. L., Lau J. Y. W. and Sung J. J. Y. (2004). Factors predicting progression of gastric intestinal metaplasia: results of a randomised trial on Helicobacter pylori eradication. *Gut* 53, 1244-1249. 10.1136/gut.2003.034629 15306578 PMC1774213

[DMM024232C91] Li Q., Karam S. M. and Gordon J. I. (1996). Diphtheria toxin-mediated ablation of parietal cells in the stomach of transgenic mice. *J. Biol. Chem.* 271, 3671-3676. 10.1074/jbc.271.7.3671 8631979

[DMM024232C92] Li H., Qi Y. and Jasper H. (2013a). Dpp signaling determines regional stem cell identity in the regenerating adult Drosophila gastrointestinal tract. *Cell Rep.* 4, 10-18. 10.1016/j.celrep.2013.05.040 23810561 PMC3778028

[DMM024232C93] Li Z., Zhang Y., Han L., Shi L. and Lin X. (2013b). Trachea-derived dpp controls adult midgut homeostasis in Drosophila. *Dev. Cell* 24, 133-143. 10.1016/j.devcel.2012.12.010 23369712

[DMM024232C94] Li N., Yousefi M., Nakauka-Ddamba A., Jain R., Tobias J., Epstein J. A., Jensen S. T. and Lengner C. J. (2014). Single-cell analysis of proxy reporter allele-marked epithelial cells establishes intestinal stem cell hierarchy. *Stem Cell Rep.* 3, 876-891. 10.1016/j.stemcr.2014.09.011 PMC4235148 25418730

[DMM024232C95] Li H., Qi Y. and Jasper H. (2016). Preventing age-related decline of gut compartmentalization limits microbiota dysbiosis and extends lifespan. *Cell Host Microbe* 19, 240-253. 10.1016/j.chom.2016.01.008 26867182 PMC5106289

[DMM024232C96] Lin G., Xu N. and Xi R. (2008). Paracrine Wingless signalling controls self-renewal of Drosophila intestinal stem cells. *Nature* 455, 1119-1123. 10.1038/nature07329 18806781

[DMM024232C97] Lin G., Xu N. and Xi R. (2010). Paracrine unpaired signaling through the JAK/STAT pathway controls self-renewal and lineage differentiation of drosophila intestinal stem cells. *J. Mol. Cell Biol.* 2, 37-49. 10.1093/jmcb/mjp028 19797317

[DMM024232C98] Liu K., Jiang M., Lu Y., Chen H., Sun J., Wu S., Ku W.-Y., Nakagawa H., Kita Y., Natsugoe S. et al. (2013). Sox2 cooperates with inflammation-mediated Stat3 activation in the malignant transformation of foregut basal progenitor cells. *Cell Stem Cell* 12, 304-315. 10.1016/j.stem.2013.01.007 23472872 PMC3594795

[DMM024232C99] Loza-Coll M. A., Southall T. D., Sandall S. L., Brand A. H. and Jones D. L. (2014). Regulation of Drosophila intestinal stem cell maintenance and differentiation by the transcription factor Escargot. *EMBO J.* 33, 2983-2996. 10.15252/embj.201489050 25433031 PMC4282644

[DMM024232C100] Madison B. B., Braunstein K., Kuizon E., Portman K., Qiao X. T. and Gumucio D. L. (2005). Epithelial hedgehog signals pattern the intestinal crypt-villus axis. *Development* 132, 279-289. 10.1242/dev.01576 15590741

[DMM024232C101] Manichanh C., Borruel N., Casellas F. and Guarner F. (2012). The gut microbiota in IBD. *Nat. Rev. Gastroenterol. Hepatol.* 9, 599-608. 10.1038/nrgastro.2012.152 22907164

[DMM024232C102] Mari L., Milano F., Parikh K., Straub D., Everts V., Hoeben K. K., Fockens P., Buttar N. S. and Krishnadath K. K. (2014). A pSMAD/CDX2 complex is essential for the intestinalization of epithelial metaplasia. *Cell Rep.* 7, 1197-1210. 10.1016/j.celrep.2014.03.074 24794431

[DMM024232C103] Marianes A. and Spradling A. C. (2013). Physiological and stem cell compartmentalization within the Drosophila midgut. *Elife* 2, e00886 10.7554/eLife.00886 23991285 PMC3755342

[DMM024232C104] Mera R., Fontham E. T. H., Bravo L. E., Bravo J. C., Piazuelo M. B., Camargo M. C. and Correa P. (2005). Long term follow up of patients treated for Helicobacter pylori infection. *Gut* 54, 1536-1540. 10.1136/gut.2005.072009 15985559 PMC1462952

[DMM024232C105] Micchelli C. A. and Perrimon N. (2006). Evidence that stem cells reside in the adult Drosophila midgut epithelium. *Nature* 439, 475-479. 10.1038/nature04371 16340959

[DMM024232C106] Milano J., McKay J., Dagenais C., Foster-Brown L., Pognan F., Gadient R., Jacobs R. T., Zacco A., Greenberg B. and Ciaccio P. J. (2004). Modulation of notch processing by gamma-secretase inhibitors causes intestinal goblet cell metaplasia and induction of genes known to specify gut secretory lineage differentiation. *Toxicol. Sci.* 82, 341-358. 10.1093/toxsci/kfh254 15319485

[DMM024232C107] Mills J. C. and Shivdasani R. A. (2011). Gastric epithelial stem cells. *Gastroenterology* 140, 412-424. 10.1053/j.gastro.2010.12.001 21144849 PMC3708552

[DMM024232C108] Mori-Akiyama Y., van den Born M., van Es J. H., Hamilton S. R., Adams H. P., Zhang J., Clevers H. and de Crombrugghe B. (2007). SOX9 is required for the differentiation of paneth cells in the intestinal epithelium. *Gastroenterology* 133, 539-546. 10.1053/j.gastro.2007.05.020 17681175

[DMM024232C109] Muncan V., Sansom O. J., Tertoolen L., Phesse T. J., Begthel H., Sancho E., Cole A. M., Gregorieff A., de Alboran I. M., Clevers H. et al. (2006). Rapid loss of intestinal crypts upon conditional deletion of the Wnt/Tcf-4 target gene c-Myc. *Mol. Cell. Biol.* 26, 8418-8426. 10.1128/MCB.00821-06 16954380 PMC1636776

[DMM024232C110] Mutoh H., Hakamata Y., Sato K., Eda A., Yanaka I., Honda S., Osawa H., Kaneko Y. and Sugano K. (2002). Conversion of gastric mucosa to intestinal metaplasia in Cdx2-expressing transgenic mice. *Biochem. Biophys. Res. Commun.* 294, 470-479. 10.1016/S0006-291X(02)00480-1 12051735

[DMM024232C168] Nagy P., Kovács L., Sándor G. O. and Juhász G. (2016). Stem-cell-specific endocytic degradation defects lead to intestinal dysplasia in Drosophila. *Dis. Model Mech.* 9, 501-512. 10.1242/dmm.023416 26921396 PMC4892661

[DMM024232C111] Nakagoshi H. (2005). Functional specification in the Drosophila endoderm. *Dev. Growth Differ.* 47, 383-392. 10.1111/j.1440-169X.2005.00811.x 16109036

[DMM024232C112] Neurath M. F. (2014). Cytokines in inflammatory bowel disease. *Nat. Rev. Immunol.* 14, 329-342. 10.1038/nri3661 24751956

[DMM024232C113] O'Brien L. E., Soliman S. S., Li X. and Bilder D. (2011). Altered modes of stem cell division drive adaptive intestinal growth. *Cell* 147, 603-614. 10.1016/j.cell.2011.08.048 22036568 PMC3246009

[DMM024232C114] Ohlstein B. and Spradling A. (2006). The adult Drosophila posterior midgut is maintained by pluripotent stem cells. *Nature* 439, 470-474. 10.1038/nature04333 16340960

[DMM024232C115] Ohlstein B. and Spradling A. (2007). Multipotent Drosophila intestinal stem cells specify daughter cell fates by differential notch signaling. *Science* 315, 988-992. 10.1126/science.1136606 17303754

[DMM024232C116] Pan Q., Nicholson A. M., Barr H., Harrison L.-A., Wilson G. D., Burkert J., Jeffery R., Alison M. R., Looijenga L., Lin W.-R. et al. (2013). Identification of lineage-uncommitted, long-lived, label-retaining cells in healthy human esophagus and stomach, and in metaplastic esophagus. *Gastroenterology* 144, 761-770. 10.1053/j.gastro.2012.12.022 23266557

[DMM024232C117] Patel P. H., Dutta D. and Edgar B. A. (2015). Niche appropriation by Drosophila intestinal stem cell tumours. *Nat. Cell Biol.* 17, 1182-1192. 10.1038/ncb3214 26237646 PMC4709566

[DMM024232C118] Piazuelo M. B., Haque S., Delgado A., Du J. X., Rodriguez F. and Correa P. (2004). Phenotypic differences between esophageal and gastric intestinal metaplasia. *Mod. Pathol.* 17, 62-74. 10.1038/modpathol.3800016 14631367

[DMM024232C119] Pinto D., Gregorieff A., Begthel H. and Clevers H. (2003). Canonical Wnt signals are essential for homeostasis of the intestinal epithelium. *Genes Dev.* 17, 1709-1713. 10.1101/gad.267103 12865297 PMC196179

[DMM024232C120] Podolsky D. K. (2002). Inflammatory bowel disease. *N. Engl. J. Med.* 347, 417-429. 10.1056/NEJMra020831 12167685

[DMM024232C121] Ponder B. A. J., Schmidt G. H., Wilkinson M. M., Wood M. J., Monk M. and Reid A. (1985). Derivation of mouse intestinal crypts from single progenitor cells. *Nature* 313, 689-691. 10.1038/313689a0 3974703

[DMM024232C122] Qiao X. T., Ziel J. W., McKimpson W., Madison B. B., Todisco A., Merchant J. L., Samuelson L. C. and Gumucio D. L. (2007). Prospective identification of a multilineage progenitor in murine stomach epithelium. *Gastroenterology* 133, 1989-1998.e3. 10.1053/j.gastro.2007.09.031 18054570 PMC2329573

[DMM024232C123] Quan Z., Sun P., Lin G. and Xi R. (2013). TSC1/2 regulates intestinal stem cell maintenance and lineage differentiation through Rheb-TORC1-S6K but independently of nutritional status or Notch regulation. *J. Cell Sci.* 126, 3884-3892. 10.1242/jcs.125294 23843608

[DMM024232C124] Quante M., Abrams J. A., Lee Y. and Wang T. C. (2012a). Barrett esophagus: what a mouse model can teach us about human disease. *Cell Cycle* 11, 4328-4338. 10.4161/cc.22485 23095673 PMC3552915

[DMM024232C125] Quante M., Bhagat G., Abrams J. A., Marache F., Good P., Lee M. D., Lee Y., Friedman R., Asfaha S., Dubeykovskaya Z. et al. (2012b). Bile acid and inflammation activate gastric cardia stem cells in a mouse model of Barrett-like metaplasia. *Cancer Cell* 21, 36-51. 10.1016/j.ccr.2011.12.004 22264787 PMC3266546

[DMM024232C126] Ren F., Wang B., Yue T., Yun E.-Y., Ip Y. T. and Jiang J. (2010). Hippo signaling regulates Drosophila intestine stem cell proliferation through multiple pathways. *Proc. Natl. Acad. Sci. USA* 107, 21064-21069. 10.1073/pnas.1012759107 21078993 PMC3000252

[DMM024232C127] Rera M., Bahadorani S., Cho J., Koehler C. L., Ulgherait M., Hur J. H., Ansari W. S., Lo T. Jr, Jones D. L. and Walker D. W. (2011). Modulation of longevity and tissue homeostasis by the Drosophila PGC-1 homolog. *Cell Metab.* 14, 623-634. 10.1016/j.cmet.2011.09.013 22055505 PMC3238792

[DMM024232C128] Rera M., Clark R. I. and Walker D. W. (2012). Intestinal barrier dysfunction links metabolic and inflammatory markers of aging to death in Drosophila. *Proc. Natl. Acad. Sci. USA* 109, 21528-21533. 10.1073/pnas.1215849110 23236133 PMC3535647

[DMM024232C129] Rugge M., Correa P., Dixon M. F., Hattori T., Leandro G., Lewin K., Riddell R. H., Sipponen P. and Watanabe H. (2000). Gastric dysplasia: the Padova international classification. *Am. J. Surg. Pathol.* 24, 167-176. 10.1097/00000478-200002000-00001 10680883

[DMM024232C130] Sangiorgi E. and Capecchi M. R. (2008). Bmi1 is expressed in vivo in intestinal stem cells. *Nat. Genet.* 40, 915-920. 10.1038/ng.165 18536716 PMC2906135

[DMM024232C131] Sarr M. G., Hamilton S. R., Marrone G. C. and Cameron J. L. (1985). Barrett's esophagus: its prevalence and association with adenocarcinoma in patients with symptoms of gastroesophageal reflux. *Am. J. Surg.* 149, 187-193. 10.1016/S0002-9610(85)80031-3 3966636

[DMM024232C132] Schepers A. and Clevers H. (2012). Wnt signaling, stem cells, and cancer of the gastrointestinal tract. *Cold Spring Harb. Perspect. Biol.* 4, a007989 10.1101/cshperspect.a007989 22474007 PMC3312683

[DMM024232C133] Siegel R., DeSantis C. and Jemal A. (2014). Colorectal cancer statistics, 2014. *CA Cancer J. Clin.* 64, 104-117. 10.3322/caac.21220 24639052

[DMM024232C134] Silberg D. G., Furth E. E., Taylor J. K., Schuck T., Chiou T. and Traber P. G. (1997). CDX1 protein expression in normal, metaplastic, and neoplastic human alimentary tract epithelium. *Gastroenterology* 113, 478-486. 10.1053/gast.1997.v113.pm9247467 9247467

[DMM024232C135] Silberg D. G., Sullivan J., Kang E., Swain G. P., Moffett J., Sund N. J., Sackett S. D. and Kaestner K. H. (2002). Cdx2 ectopic expression induces gastric intestinal metaplasia in transgenic mice. *Gastroenterology* 122, 689-696. 10.1053/gast.2002.31902 11875002

[DMM024232C136] Siudeja K., Nassari S., Gervais L., Skorski P., Lameiras S., Stolfa D., Zande M., Bernard V., Rio Frio T. and Bardin A. J. (2015). Frequent somatic mutation in adult intestinal stem cells drives neoplasia and genetic mosaicism during aging. *Cell Stem Cell* 17, 663-674. 10.1016/j.stem.2015.09.016 26607382 PMC5138153

[DMM024232C137] Sivan A., Corrales L., Hubert N., Williams J. B., Aquino-Michaels K., Earley Z. M., Benyamin F. W., Lei Y. M., Jabri B., Alegre M.-L. et al. (2015). Commensal Bifidobacterium promotes antitumor immunity and facilitates anti-PD-L1 efficacy. *Science* 350, 1084-1089. 10.1126/science.aac4255 26541606 PMC4873287

[DMM024232C138] Slack J. M. W. (2007). Metaplasia and transdifferentiation: from pure biology to the clinic. *Nat. Rev. Mol. Cell Biol.* 8, 369-378. 10.1038/nrm2146 17377526

[DMM024232C139] Staley B. K. and Irvine K. D. (2010). Warts and Yorkie mediate intestinal regeneration by influencing stem cell proliferation. *Curr. Biol.* 20, 1580-1587. 10.1016/j.cub.2010.07.041 20727758 PMC2955330

[DMM024232C140] Stange D. E., Koo B.-K., Huch M., Sibbel G., Basak O., Lyubimova A., Kujala P., Bartfeld S., Koster J., Geahlen J. H. et al. (2013). Differentiated Troy+ chief cells act as reserve stem cells to generate all lineages of the stomach epithelium. *Cell* 155, 357-368. 10.1016/j.cell.2013.09.008 24120136 PMC4094146

[DMM024232C141] Strand M. and Micchelli C. A. (2011). Quiescent gastric stem cells maintain the adult Drosophila stomach. *Proc. Natl. Acad. Sci. USA* 108, 17696-17701. 10.1073/pnas.1109794108 21984734 PMC3203805

[DMM024232C142] Strand M. and Micchelli C. A. (2013). Regional control of Drosophila gut stem cell proliferation: EGF establishes GSSC proliferative set point & controls emergence from quiescence. *PLoS ONE* 8, e80608 10.1371/journal.pone.0080608 24236188 PMC3827418

[DMM024232C143] Takashima S., Mkrtchyan M., Younossi-Hartenstein A., Merriam J. R. and Hartenstein V. (2008). The behaviour of Drosophila adult hindgut stem cells is controlled by Wnt and Hh signalling. *Nature* 454, 651-655. 10.1038/nature07156 18633350

[DMM024232C144] Tian A. and Jiang J. (2014). Intestinal epithelium-derived BMP controls stem cell self-renewal in Drosophila adult midgut. *Elife* 3, e01857 10.7554/eLife.01857 24618900 PMC3948108

[DMM024232C145] Tian H., Biehs B., Warming S., Leong K. G., Rangell L., Klein O. D. and de Sauvage F. J. (2011). A reserve stem cell population in small intestine renders Lgr5-positive cells dispensable. *Nature* 478, 255-259. 10.1038/nature10408 21927002 PMC4251967

[DMM024232C146] Tian H., Biehs B., Chiu C., Siebel C. W., Wu Y., Costa M., de Sauvage F. J. and Klein O. D. (2015). Opposing activities of Notch and Wnt signaling regulate intestinal stem cells and gut homeostasis. *Cell Rep.* 11, 33-42. 10.1016/j.celrep.2015.03.007 25818302 PMC4394041

[DMM024232C147] Uemura N., Okamoto S., Yamamoto S., Matsumura N., Yamaguchi S., Yamakido M., Taniyama K., Sasaki N. and Schlemper R. J. (2001). Helicobacter pylori infection and the development of gastric cancer. *N. Engl. J. Med.* 345, 784-789. 10.1056/NEJMoa001999 11556297

[DMM024232C148] Ullman T., Odze R. and Farraye F. A. (2009). Diagnosis and management of dysplasia in patients with ulcerative colitis and Crohn's disease of the colon. *Inflamm. Bowel Dis.* 15, 630-638. 10.1002/ibd.20766 18942763 PMC2753500

[DMM024232C149] Uronis J. M., Muhlbauer M., Herfarth H. H., Rubinas T. C., Jones G. S. and Jobin C. (2009). Modulation of the intestinal microbiota alters colitis-associated colorectal cancer susceptibility. *PLoS ONE* 4, e6026 10.1371/journal.pone.0006026 19551144 PMC2696084

[DMM024232C150] van de Wetering M., Sancho E., Verweij C., de Lau W., Oving I., Hurlstone A., van der Horn K., Batlle E., Coudreuse D., Haramis A.-P. et al. (2002). The beta-catenin/TCF-4 complex imposes a crypt progenitor phenotype on colorectal cancer cells. *Cell* 111, 241-250. 10.1016/S0092-8674(02)01014-0 12408868

[DMM024232C151] van der Flier L. G. and Clevers H. (2009). Stem cells, self-renewal, and differentiation in the intestinal epithelium. *Annu. Rev. Physiol.* 71, 241-260. 10.1146/annurev.physiol.010908.163145 18808327

[DMM024232C152] van Dop W. A., Uhmann A., Wijgerde M., Sleddens-Linkels E., Heijmans J., Offerhaus G. J., van den Bergh Weerman M. A., Boeckxstaens G. E., Hommes D. W., Hardwick J. C. et al. (2009). Depletion of the colonic epithelial precursor cell compartment upon conditional activation of the hedgehog pathway. *Gastroenterology* 136, 2195-2203 e2191-2197 10.1053/j.gastro.2009.02.068 19272384

[DMM024232C153] van Es J. H., Jay P., Gregorieff A., van Gijn M. E., Jonkheer S., Hatzis P., Thiele A., van den Born M., Begthel H., Brabletz T. et al. (2005a). Wnt signalling induces maturation of Paneth cells in intestinal crypts. *Nat. Cell Biol.* 7, 381-386. 10.1038/ncb1240 15778706

[DMM024232C154] van Es J. H., van Gijn M. E., Riccio O., van den Born M., Vooijs M., Begthel H., Cozijnsen M., Robine S., Winton D. J., Radtke F. et al. (2005b). Notch/gamma-secretase inhibition turns proliferative cells in intestinal crypts and adenomas into goblet cells. *Nature* 435, 959-963. 10.1038/nature03659 15959515

[DMM024232C155] Vetizou M., Pitt J. M., Daillere R., Lepage P., Waldschmitt N., Flament C., Rusakiewicz S., Routy B., Roberti M. P., Duong C. P. M. et al. (2015). Anticancer immunotherapy by CTLA-4 blockade relies on the gut microbiota. *Science* 350, 1079-1084. 10.1126/science.aad1329 26541610 PMC4721659

[DMM024232C156] von Rahden B. H. A., Kircher S., Lazariotou M., Reiber C., Stuermer L., Otto C., Germer C. T. and Grimm M. (2011). LgR5 expression and cancer stem cell hypothesis: clue to define the true origin of esophageal adenocarcinomas with and without Barrett's esophagus? *J. Exp. Clin. Cancer Res.* 30, 23 10.1186/1756-9966-30-23 21345220 PMC3058063

[DMM024232C157] Walker M. R., Patel K. K. and Stappenbeck T. S. (2009). The stem cell niche. *J. Pathol.* 217, 169-180. 10.1002/path.2474 19089901

[DMM024232C158] Wang X., Ouyang H., Yamamoto Y., Kumar P. A., Wei T. S., Dagher R., Vincent M., Lu X., Bellizzi A. M., Ho K. Y. et al. (2011). Residual embryonic cells as precursors of a Barrett's-like metaplasia. *Cell* 145, 1023-1035. 10.1016/j.cell.2011.05.026 21703447 PMC3125107

[DMM024232C159] Wang C., Guo X. and Xi R. (2014). EGFR and Notch signaling respectively regulate proliferative activity and multiple cell lineage differentiation of Drosophila gastric stem cells. *Cell Res.* 24, 610-627. 10.1038/cr.2014.27 24603358 PMC4011342

[DMM024232C160] Wang C., Guo X., Dou K., Chen H. and Xi R. (2015a). Ttk69 acts as a master repressor of enteroendocrine cell specification in Drosophila intestinal stem cell lineages. *Development* 142, 3321-3331. 10.1242/dev.123208 26293304

[DMM024232C161] Wang L., Ryoo H. D., Qi Y. and Jasper H. (2015b). PERK limits Drosophila lifespan by promoting intestinal stem cell proliferation in response to ER stress. *PLoS Genet.* 11, e1005220 10.1371/journal.pgen.1005220 25945494 PMC4422665

[DMM024232C162] Wilson K. T. and Crabtree J. E. (2007). Immunology of Helicobacter pylori: insights into the failure of the immune response and perspectives on vaccine studies. *Gastroenterology* 133, 288-308. 10.1053/j.gastro.2007.05.008 17631150

[DMM024232C163] Winters C. Jr, Spurling T. J., Chobanian S. J., Curtis D. J., Esposito R. L., Hacker J. F. III, Johnson D. A., Cruess D. F., Cotelingam J. D., Gurney M. S. et al. (1987). Barrett's esophagus. A prevalent, occult complication of gastroesophageal reflux disease. *Gastroenterology* 92, 118-124. 3781178

[DMM024232C164] Wong G. T., Manfra D., Poulet F. M., Zhang Q., Josien H., Bara T., Engstrom L., Pinzon-Ortiz M., Fine J. S., Lee H.-J. J. et al. (2004). Chronic treatment with the gamma-secretase inhibitor LY-411,575 inhibits beta-amyloid peptide production and alters lymphopoiesis and intestinal cell differentiation. *J. Biol. Chem.* 279, 12876-12882. 10.1074/jbc.M311652200 14709552

[DMM024232C165] Yan K. S., Chia L. A., Li X., Ootani A., Su J., Lee J. Y., Su N., Luo Y., Heilshorn S. C., Amieva M. R. et al. (2012). The intestinal stem cell markers Bmi1 and Lgr5 identify two functionally distinct populations. *Proc. Natl. Acad. Sci. USA* 109, 466-471. 10.1073/pnas.1118857109 22190486 PMC3258636

[DMM024232C166] Yeung T. M., Chia L. A., Kosinski C. M. and Kuo C. J. (2011). Regulation of self-renewal and differentiation by the intestinal stem cell niche. *Cell. Mol. Life Sci.* 68, 2513-2523. 10.1007/s00018-011-0687-5 21509540 PMC4165857

[DMM024232C167] Zeng X. and Hou S. X. (2015). Enteroendocrine cells are generated from stem cells through a distinct progenitor in the adult Drosophila posterior midgut. *Development* 142, 644-653. 10.1242/dev.113357 25670791 PMC4325374

